# Potentials of ChatGPT in Anatomy Research: A Conversation With ChatGPT

**DOI:** 10.7759/cureus.94984

**Published:** 2025-10-20

**Authors:** Sarika R Tigga, Sandeep Saluja

**Affiliations:** 1 Anatomy, University College of Medical Sciences, Guru Teg Bahadur Hospital, New Delhi, IND; 2 Anatomy, Amrita School of Medicine, Faridabad, IND

**Keywords:** anatomy, anatomy research, artificial intelligence, chatbot, chatgpt

## Abstract

Background: The field of natural language processing (NLP) has experienced considerable growth in recent times, advancing towards the capability of human-like language production. Among these developments, ChatGPT (OpenAI, San Francisco, CA, USA) is an excellent language model efficient in producing rational and contextually pertinent text responses. Its capability to comprehend and react to user inputs has created new opportunities for a wide range of implementations, encompassing research and publication. This study intended to evaluate the capabilities of ChatGPT and its optimal utilisation in anatomy research (AR).

Methodology: The study included 22 prompts, which were submitted to ChatGPT after obtaining an online subscription to the Plus plan. These prompts were formulated after consensus among the investigators. The chatbot's responses were documented and assessed with regard to relevance, precision, and reliability.

Results: ChatGPT was observed to be helpful for the investigators to discover the potential topics for exploration in anatomy and recognise research gaps in existing literature. The chatbot created a good synopsis of an article and wrote a satisfactory literature review and research proposal on the presented topic. It was noted to provide adequate assistance to the researchers in the preparation of the manuscript and was quite capable of suggesting the applicable statistical test for any given data. The chatbot also performed various statistical tests and provided assistance in the graphical visualisation of the data by drawing histograms, box plots, and scatter plots.

Conclusions: ChatGPT can be a valuable interactive tool for the investigators with the capacity to play a fundamental role in AR if utilised methodically. It provides considerable assistance to the anatomy researchers during the selection of research topics, writing of research proposals, preparation of the manuscript, and carrying out of statistical analysis.

## Introduction

Artificial intelligence (AI) is an extensive domain within computer science that refers to the emulation of human intelligence by a machine or system [1,2]. The objective of AI is to evolve a machine that can think like humans and mimic human behaviours, including the capability to comprehend, transform, rationalise, recognise, design, predict, anticipate, and understand theoretical concepts as well [1,3]. ChatGPT (created by OpenAI, San Francisco, CA, USA) is an AI-based large language model (LLM) trained on vast multilingual text data sets, intended to produce human-like responses to textual prompts [3,4]. ChatGPT, as a natural language processing (NLP) tool, has a significant impact, and its ongoing development is poised to reshape the field of conversational AI across multiple areas. In the healthcare sector, professionals are increasingly leveraging AI technologies like ChatGPT to increase productivity in areas such as diagnosis, medical imaging interpretation, predictive models, and personalised treatment techniques. ChatGPT holds the capability to improve these applications by incorporating its conversational abilities with medical expertise [5,6]. Its ability to convey clear and pertinent information underlines its value as a promising tool for medical research as well. It can support healthcare professionals by condensing recent medical research from extensive databases [4].

The advent of ChatGPT has markedly reshaped the conventional approach to research and publishing scientific papers. Investigators and authors can utilise this advanced language model to optimise their workflow, resulting in greater productivity and enhanced quality of publications. ChatGPT can be a valuable aid throughout the research writing process, helping in generating titles, summarising complex information into clear abstracts, assisting in literature reviews, conceptualising methodologies, and developing comprehensive discussions and well-rounded conclusions. By providing key study details, researchers can effectively collaborate with ChatGPT to enhance the clarity and impact of their research work [7].

However, effective application of ChatGPT in the research and publication sector demands a thorough awareness of its strengths, limitations, and recommended usage practices [7]. Although ChatGPT offers huge potential, it still encounters challenges like authorship issues, data errors, unpredictability, variability, interpretability, inherent biases, and apprehensions regarding plagiarism. It is essential to tackle ethical and legal issues while ensuring academic integrity and the credibility of data [7,8]. Medical publications offer healthcare professionals valuable insights into innovative treatments and advanced diagnostic approaches. Hence, it is crucial to meticulously evaluate the potentials of AI tools like ChatGPT when drafting healthcare articles [9]. 

Anatomy continues to be a pillar of medical science. A thorough knowledge of anatomy is essential to grasp both basic and clinical subjects related to the investigation of disease. In addition to its critical role in diagnosis, anatomy is equally important in the treatment process. Furthermore, a wide range of anaesthetic procedures and surgical interventions necessitate a comprehensive anatomical knowledge of the various structures of the human body [10]. In this context, we aim to evaluate the potentials of ChatGPT for conducting research in human anatomy. The present study introduced a few prompts to ChatGPT-5 to investigate its current capabilities and best possible application in anatomy research (AR) and publication.

## Materials and methods

In the present study, ChatGPT (5th version) was employed, which is regarded as superior to earlier versions. A range of prompts were presented to ChatGPT to evaluate its potential in AR. Following the initial query about ChatGPT's role in AR, various prompts were submitted. Initial prompts were based on the responses generated by ChatGPT. Further prompts were submitted to evaluate its ability to draft various sections of a scientific paper. These prompts challenged the chatbot to manifest its capacity to offer the topics of potential research in anatomy, compile and summarise the key findings of necessary articles, and write literature reviews, research proposals, and references, as well as its ability to do statistical analysis and graphical representation of the data. To evaluate ChatGPT's capability for calculating the mean and standard deviation, we submitted the length of 108 dry adult human sacra in the Excel file as a data set in a prompt. Further, the heights of the right and left first posterior sacral foramina of the sacra were presented to the chatbot in the Excel file as data sets to assess its potential for determining the paired t-test and Pearson correlation coefficient, as well as the generation of a histogram, box plot, and scatter plot. All the prompts were formulated after mutual agreement among the researchers (Table 1). No prompt was submitted to find out the chatbot's capability to generate a completely fake original research article, as the authors agreed to investigate only its beneficial applications.

**Table 1:** Prompts submitted to ChatGPT. AI: artificial intelligence; LLM: large language model; MeSH: Medical Subject Headings

Prompt number	Questions
1	As an AI-based LLM, describe your role in anatomy research and publication.
2	Please suggest a few topics of potential research in gross anatomy.
3	What were your criteria for selecting these topics?
4	Can you compile a list of articles with DOI published in high-impact journals over the last five years that focus on the topic of "Anatomical variations of the Circle of Willis and their correlation with cerebrovascular disease risk"?
5	Please tabulate the key findings of all the above-mentioned articles in five columns: authors' names, publication year, study population, imaging technique used, and summary/key findings.
6	Can you summarise the article titled "Association between Anatomical Variations of the Circle of Willis and Covert Vascular Brain Injury in the General Population" in 500 words?
7	Please write a literature review with references on the topic of "Anatomical variations of the Circle of Willis and their correlation with cerebrovascular disease risk".
8	Can you write about the research gaps on the topic of "Anatomical variations of the Circle of Willis and their correlation with cerebrovascular disease risk"?
9	Please write a research proposal on the topic of "Anatomical variations of the Circle of Willis and their correlation with cerebrovascular disease risk".
10	Can you write an introduction with references on the topic of "Anatomical variations of the Circle of Willis and their correlation with cerebrovascular disease risk" in 500 words?
11	Please write materials and methods in detail on the topic of "Anatomical variations of the Circle of Willis and their correlation with cerebrovascular disease risk".
12	Can you please suggest salient points to write in the results, discussion, and conclusions on the topic of "Anatomical variations of the Circle of Willis and their correlation with cerebrovascular disease risk"?
13	Can you do the statistical analysis of any given data?
14	Please calculate the mean and standard deviation for the data uploaded in the Excel file.
15	Please do the paired t-test for the data uploaded in the Excel file.
16	Please calculate the Pearson correlation coefficient for the data uploaded in the Excel file.
17	Can you guide the researcher about the applicable statistical test for the data uploaded in the Excel file?
18	Can you make a histogram for the data uploaded in the Excel file?
19	Please make a box plot for the data uploaded in the Excel file.
20	Can you draw a scatter plot for the data uploaded in the Excel file?
21	Please write the reference in Vancouver style with DOI for the article titled "Association between Anatomical Variations of the Circle of Willis and Covert Vascular Brain Injury in the General Population”.
22	Can you suggest five keywords and MeSH terms for the text uploaded in the MS Word file?

The responses produced by ChatGPT were documented in their original form without any alteration. All the answers generated by the chatbot were checked for any plagiarism with the help of the "Quetext" online plagiarism detection tool. Both researchers evaluated the responses collectively for their relevance, accuracy, and comprehensiveness with the holistic rubric evaluation method using predetermined criteria and gave an independent score (1-4) to all answers to ChatGPT (Table 2). The average score for each response and the total average score for all ChatGPT responses were calculated.

**Table 2:** Holistic rubric score (1-4) criteria for ChatGPT's responses.

Level	Score	Criteria
Excellent	4	The response of ChatGPT was perfectly relevant, accurate, and comprehensive
Good	3	The response of ChatGPT was relevant, accurate, and less comprehensive
Satisfactory	2	The response of ChatGPT was somewhat relevant, partially correct, and less comprehensive
Unsatisfactory	1	The response of ChatGPT was irrelevant, inaccurate, and restricted

## Results

A total of 22 prompts were submitted, and the responses generated by ChatGPT-5 were recorded without doing any modification (Table 3).

**Table 3:** Responses generated by ChatGPT.

Prompt number	Responses
1	As an AI-based large language model (LLM), my role in anatomy research and publication spans multiple stages of the scientific process: Topic Selection & Idea Generation – Identifying novel and relevant research areas in anatomy by analyzing current literature, detecting gaps, and suggesting clinically significant directions. Literature Review & Evidence Synthesis – Conducting rapid, targeted searches, summarizing key findings, and organizing evidence for narrative, scoping, or systematic reviews. Study Design Support – Advising on research methodologies, anatomical study models (cadaveric, imaging-based, morphometric), sampling strategies, and ethical considerations. Data Handling & Analysis Guidance – Assisting in selecting appropriate statistical methods, explaining their application, and generating descriptive/graphical outputs for anatomical data. Manuscript Preparation – Drafting and refining sections such as introductions, methods, results, and discussions; ensuring clarity, coherence, and academic tone. Illustrations & Figures – Creating clear, labeled anatomical diagrams, flowcharts, and tables for effective visual communication. Editing & Proofreading – Checking grammar, terminology, and logical flow; ensuring adherence to journal-specific guidelines and referencing styles (e.g., Vancouver, APA). Reference Management – Formatting and verifying citations, cross-checking DOIs, and ensuring bibliographic accuracy. By integrating speed, language precision, and access to a vast knowledge base, I function as a research assistant, language editor, and knowledge synthesizer—enhancing the efficiency and quality of anatomy research and publication while complementing, not replacing, the expertise of human anatomists and researchers.
2	Here are several potential research topics in gross anatomy that align with current trends, knowledge gaps, and clinical relevance: 1. Neuroanatomy & Neurosurgical Relevance Anatomical variations of the Circle of Willis and their correlation with cerebrovascular disease risk. Morphometric analysis of the optic nerve and canal in relation to surgical decompression. Anatomical study of cranial nerve exit foramina and implications for skull base surgery. 2. Cardiovascular & Thoracic Anatomy Variations in the branching pattern of the aortic arch in different populations. Morphology and clinical significance of myocardial bridges on coronary arteries. Anatomical correlations between pericardial sinuses and cardiac surgical approaches. 3. Musculoskeletal Anatomy Morphometric study of acetabular depth and orientation in relation to hip dysplasia. Anatomical variations in the insertion of rotator cuff tendons and their surgical implications. Morphology of plantar fascia in relation to plantar fasciitis and foot arch types. 4. Abdominal & Pelvic Anatomy Variations in celiac trunk branching patterns and implications for hepatobiliary surgery. Anatomical relationships of the ureter with pelvic vessels and surgical safety zones. Morphometry of the inguinal canal and its correlation with hernia risk. 5. Head & Neck Anatomy Anatomical variations in the facial artery course relevant to maxillofacial surgery. Morphology of the styloid process in Eagle’s syndrome. Morphometric analysis of paranasal sinuses using CT imaging and its surgical relevance. 6. Forensic & Anthropological Anatomy Correlation of craniofacial measurements with sex determination in a specific population. Anatomical basis for age estimation from rib morphology in forensic cases. Relationship between long bone morphometry and stature estimation in different ethnic groups. If you’d like, I can narrow these down to 5 high-impact topics that are most likely to get international publication acceptance based on current anatomy research trends and journal preferences. That way, you’ll have a shortlist that’s both feasible and in-demand.
3	I selected these gross anatomy research topics using criteria that balance academic novelty, clinical relevance, and feasibility, specifically: 1. Clinical Relevance Chose topics where anatomical findings directly impact diagnosis, surgical safety, or treatment outcomes. Example: Anatomical variations of the Circle of Willis—highly relevant for stroke risk and neurosurgery. 2. Research Gap & Novelty Prioritized areas where recent studies suggest limited or region-specific data. Example: Celiac trunk branching patterns—well-studied in some regions but lacking population-specific morphometric data in others. 3. Feasibility Selected topics that can be investigated using cadaveric dissection, imaging (CT/MRI), or morphometric analysis without requiring excessive resources. Ensures accessibility for most anatomy departments. 4. Publication Potential Chose subjects frequently published in reputable anatomy, surgery, and radiology journals but still open for region-specific or methodological contributions. Example: Rotator cuff tendon insertion variations—of interest to orthopedic and sports medicine journals. 5. Interdisciplinary Appeal Focused on topics that can attract multidisciplinary collaboration (radiology, surgery, forensic science, anthropology). Broadens readership and citation potential. 6. Quantifiable Outcomes Preference for topics allowing objective measurements (angles, lengths, diameters, prevalence rates) for stronger statistical analysis. If you want, I can now rank the earlier list by “high clinical impact + high publication chance” so you’ll know which topics could get the best research and journal acceptance outcomes.
4	Absolutely—here’s a concise, vetted list from the past five years (Aug 2020–Aug 2025) in high-impact neurology/neuroradiology/stroke journals, each with a DOI: Nanninga MHA, van Strien DAM, Velthuis BK, et al. Sex-Specific Association between Anatomic Variants of the Circle of Willis and Intracranial Aneurysms in Individuals with a Familial Predisposition. *AJNR Am J Neuroradiol.* 2025;46(8):1542-1547. DOI: 10.3174/ajnr.A8690. PubMed Wang H, Shen L, Zhao C, et al. The incomplete circle of Willis is associated with vulnerable intracranial plaque features and acute ischemic stroke. *J Cardiovasc Magn Reson.* 2023;25(1):23. DOI: 10.1186/s12968-023-00931-2. PubMed Westphal LP, Lohaus N, Winklhofer S, et al. Circle of Willis variants and their association with outcome in patients with middle cerebral artery–M1-occlusion stroke. *Eur J Neurol.* 2021;28(11):3682-3691. DOI: 10.1111/ene.15013. PMC Lin E, Kamel H, Gupta A, et al. Incomplete circle of Willis variants and stroke outcome. *Eur J Radiol.* 2022;153:110383. DOI: 10.1016/j.ejrad.2022.110383. PubMed Feng L, Zhai F-F, Li M-L, et al. Association between Anatomical Variations of the Circle of Willis and Covert Vascular Brain Injury in the General Population. *Cerebrovasc Dis.* 2023;52(4):480-486. DOI: 10.1159/000527432. PubMed Hindenes LB, Nordby J, Isaksen JG, et al. Anatomical variations and incompleteness of the Circle of Willis are associated with increased odds of intracranial aneurysms: The Tromsø Study. *J Neurol Sci.* 2023;449:120634. DOI: 10.1016/j.jns.2023.120634. BioMed Central Hindenes LB, Håberg AK, Mathiesen EB, Vangberg TR. An incomplete Circle of Willis is not a risk factor for white matter hyperintensities: The Tromsø Study. *J Neurol Sci.* 2021;420:117268. DOI: 10.1016/j.jns.2020.117268. PubMedOUCI De Caro J, Ciacciarelli A, Tessitore A, et al. Variants of the circle of Willis in ischemic stroke patients. *J Neurol.* 2021;268(10):3799-3807. DOI: 10.1007/s00415-021-10454-4. PubMed Rangus I, Milles LS, Galinovic I, et al. Reclassifications of ischemic stroke patterns due to variants of the Circle of Willis. *Int J Stroke.* 2022;17(7):770-776. DOI: 10.1177/17474930211048381. PubMed Notes • I prioritized Q1 journals or widely cited specialty journals (AJNR, *JCMR*, *Eur J Neurol*, *J Neurol*, *Int J Stroke*, *Cerebrovascular Diseases*). • Items span aneurysm risk, acute ischemic stroke outcomes, plaque vulnerability, and covert small-vessel disease—each directly linking Circle of Willis (CoW) variants to cerebrovascular risk or its clinical consequences. • If you’d like, I can export these to a formatted reference list (Vancouver/APA) or build a table with study design, sample, imaging, and main findings.
5	Here’s a compact table of the key details from the articles we identified earlier. Authors Year Study population Imaging technique Summary / key findings Nanninga MHA, van Strien DAM, Velthuis BK, Velthuis MRE, Vos IN, Groenheide PJ, Ruigrok YM, van der Schaaf IC 2025 1,291 adults with familial predisposition to aSAH, screened for aneurysm Screening MRA (time-of-flight) Normal anterior CoW variant was less common in those with aneurysm (overall OR≈0.59), with a sex-specific effect in women; ACoA hypoplasia/absence more frequent in aneurysm carriers; no differences for posterior CoW variants. PubMed Wang H, Shen L, Zhao C, Liu S, Wu G, et al. 2023 97 patients with AIS/TIA and ICAS, imaged within 7 days of symptom onset 3T high-resolution vessel-wall CMR (pre/post contrast) plus TOF/CMRA Incomplete anterior CoW associated with more “vulnerable” culprit plaque features (higher enhancement ratio/grade and normalized wall index); incomplete ipsilateral posterior CoW linked to high T1 signal; features associated with AIS vs TIA. BioMed Central+1 Westphal LP, Lohaus N, Winklhofer S, et al. 2021 193 MCA-M1 occlusion stroke patients treated with EVT + 73 TIA controls TOF-MRA (stroke and TIA cohorts) CoW variants common (especially posterior) but not independently associated with 3-month functional outcome after EVT (no outcome difference after adjustment). PMC Lin E, Kamel H, Gupta A, RoyChoudhury A, Girgis P, Glodzik L 2022 297 consecutive acute stroke patients MRA on admission (reviewed for A1/P1 aplasia/hypoplasia; vertebral variants) Incomplete CoW in 34%; independently associated with lower odds of good functional status at discharge (OR≈0.53 after adjusting for age and admission NIHSS). PubMedPMC Feng L, Mao H-J, Zhang D-D, Zhu Y-C, Han F 2023 1,055 community-dwelling adults (general population) Brain MRI with MRA (3T) Incomplete/variant CoW configurations correlated with markers of covert vascular brain injury (e.g., covert infarcts, WMH/microbleeds), suggesting higher subclinical cerebrovascular risk burden. PMC Hindenes LB, Ingebrigtsen T, Isaksen JG, et al. (Tromsø Study) 2023 1,667 adults (40–84 y) from population sample 3T TOF-MRA Incomplete CoW associated with higher prevalence of intracranial aneurysm in the general population (overall OR≈2.3); strongest signals for variants lacking all communicating arteries or missing P1 segment. PubMed Hindenes LB, Haberg AK, Johnsen L-H, et al. (Tromsø) 2021 1,751 adults from population sample 3T TOF-MRA No evidence that incomplete CoW variants are associated with total WMH burden in the general population, tempering links to chronic small-vessel disease markers. PubMed De Caro J, Ciacciarelli A, Tessitore A, et al. 2021 1,131 ischemic stroke patients vs 562 age/sex-matched controls Clinical vascular imaging (MRA/CTA; neuroradiologist review) CoW variants more prevalent in stroke patients than controls, independent of large-artery occlusion; variants did not significantly influence stroke severity or in-hospital prognosis. PubMedDOI Resolver Rangus I, Milles LS, Galinovic I, et al. 2022 1,000 acute ischemic stroke patients (1000+ study) TOF-MRA vCoW present in ~37%; fetal-type PCA often mimicked multi-territory patterns—reclassification from “>1 territory” to “1 territory” in ~8% once variants considered; highlights diagnostic/etiologic implications. PubMed If you’d like, I can export this as a CSV/Excel file or add extra columns (e.g., CoW classification scheme used, primary outcomes, adjusted covariates).
6	Here’s a 500‑word summary of the article “Association between Anatomical Variations of the Circle of Willis and Covert Vascular Brain Injury in the General Population” by Lu Feng et al. (2023): Background & Purpose The Circle of Willis (CoW) is a vital arterial ring at the brain’s base, acting as a key collateral circulation system. While anatomical variants are common and typically asymptomatic, they may diminish the CoW’s ability to compensate for impaired cerebral blood flow, potentially leading to covert (silent) vascular brain injury. Such injuries—white matter hyperintensities (WMHs), lacunes, microbleeds, enlarged perivascular spaces (EPVS), and brain atrophy—are often subclinical but known risk factors for stroke, cognitive decline, and dementia. Despite this, the association between CoW anatomical variation and covert vascular brain injury in the general population remained underexplored.MDPI+15PubMed+15ResearchGate+15 Study Population & Methods This cross-sectional study analyzed data from 1,055 community-dwelling Chinese adults (mean age 54.8 ± 8.9 years; 36% men) without intracranial arterial stenosis. Participants were drawn from the Shunyi Study cohort, recruited between 2013 and 2014. Brain imaging included MRI to detect covert vascular lesions and MR angiography (MRA) to assess CoW configurations, focusing on completeness, symmetry, and presence of the fetal-type posterior cerebral artery (FTP).ResearchGate+1 Key Findings Only 104 participants (9.9%) had a complete CoW. Completeness decreased with age (p = 0.001), indicating that anatomical variation increases over the lifespan.BioMed Central+15PubMed+15ResearchGate+15 Incomplete CoW significantly correlated with more severe WMHs (odds ratio [OR] = 2.071; 95% CI: 1.004–4.270) and greater prevalence of cerebral microbleeds (CMBs) (OR = 1.542; 95% CI: 1.012–2.348), after adjusting for age and sex.PubMed+2ResearchGate+2 Fetal-type PCA (FTP) presence also showed significant associations with several covert lesions: Lacunes: OR = 1.878 (95% CI: 1.069–3.298) More severe WMHs: OR = 1.739 (95% CI: 1.064–2.842) Less severe enlarged perivascular spaces (EPVS): OR = 0.562 (95% CI: 0.346–0.915) This suggests a complex relationship in which FTP may heighten risk for some lesions (lacunes, WMH) while protecting against others (EPVS).MDPI+12PubMed+12ResearchGate+12 Interpretation & Discussion The findings underscore that CoW configuration significantly influences susceptibility to silent cerebrovascular damage in a general population. An incomplete CoW may restrict collateral flow, thereby exacerbating chronic, subclinical ischemic injury. FTP presence likewise alters perfusion dynamics—perhaps increasing risk of lacunar and white matter lesions while reducing EPVS through different hemodynamic mechanisms. The study also emphasizes the importance of symmetry and regional completeness (anterior versus posterior CoW), suggesting that asymmetries may impair perfusion more than previously appreciated. For instance, asymmetry in anterior circulation correlated with greater brain atrophy, while posterior incompleteness aligned more closely with WMH severity.Karger+3ResearchGate+3BioMed Central+3 Limitations & Future Directions This cross-sectional analysis precludes causal inference. Moreover, the study did not consider lesion laterality relative to CoW variants due to limited lesion numbers, which may obscure side-specific effects. Future longitudinal studies should clarify causal pathways and explore whether CoW-based risk stratification could help in preventive neurology. Conclusion Anatomical variations of the CoW—including incomplete configuration and presence of FTP—are significantly associated with multiple forms of covert vascular brain injury. These findings suggest that detailed vascular anatomy may serve as a biomarker for subclinical vascular brain damage and warrant further study in longitudinal and clinical-risk models.PubMed+2ResearchGate+2
7	Anatomical variations of the Circle of Willis and correlation with cerebrovascular disease risk: a literature review (2020–2025) Introduction The Circle of Willis (CoW) provides potential collateral routes between the anterior and posterior cerebral circulations. Variability is common—hypoplasia or aplasia of the communicating segments, fetal-type posterior cerebral artery (fPCA), and asymmetric A1 segments are among the frequent patterns. Interest in these variants has grown because collateral adequacy and local hemodynamics are central to the risk and phenotype of cerebrovascular disease (CVD), including ischemic stroke, intracranial aneurysm, and covert small vessel disease. This review synthesizes recent evidence (roughly the last five years) linking CoW anatomy to CVD risk and outcomes. Imaging approaches and classification Modern studies classify CoW configurations on noninvasive angiography—most often 3D time-of-flight MR angiography (TOF-MRA) or CT angiography (CTA)—and, in disease-focused cohorts, add intracranial vessel-wall MRI to interrogate plaque morphology. These methods allow standardized assessment of “completeness,” segment hypoplasia, and presence of fPCA, and they increasingly enable linkage to downstream parenchymal injury on structural MRI. BioMed Central Covert (subclinical) vascular brain injury A population-based study from the Shunyi cohort evaluated 1,055 community-dwelling adults with MRA for CoW configuration and brain MRI for markers of covert cerebrovascular injury (white-matter hyperintensities, lacunes, microbleeds, enlarged perivascular spaces). Incomplete CoW and fPCA were associated with a higher burden of several covert injury markers after adjustment for confounders, supporting the concept that variant anatomy predisposes to subclinical cerebrovascular damage via impaired collateralization or altered flow partitioning. PMC That said, results are not uniform across populations and phenotypes. In the Tromsø Study, an earlier analysis focusing on white-matter hyperintensities alone found no association between incomplete CoW and WMH volume after risk-factor adjustment, underscoring phenotype- and cohort-specific effects and the importance of comprehensive lesion panels. PubMed Intracranial aneurysm risk A large, population-based analysis from the Tromsø Study linked CoW variants on TOF-MRA with the presence of intracranial aneurysms (IAs). Incomplete configurations (notably absent/hypoplastic communicating segments and certain posterior variants) were associated with higher odds of unruptured IAs in multivariable models, consistent with hemodynamic stress concentration at bifurcations supplied by altered inflow. PubMedJNS Journal+1ScienceDirect Complementing this, a systematic review focusing on aneurysms at the anterior and posterior communicating complexes suggests that specific CoW topologies may influence both formation and rupture propensity at these sites, although heterogeneity of definitions remains a limitation. Frontiers Large-artery atherosclerosis and acute ischemic stroke (AIS) Linking anatomy to mechanism, a vessel-wall MRI study in patients with intracranial atherosclerosis reported that incomplete CoW configurations were associated with higher-risk intracranial plaque features (e.g., positive remodeling, T1 hyperintensity) and with acute ischemic stroke, suggesting that deficient collateral supply may interact with plaque vulnerability and downstream perfusion compromise. BioMed CentralPMCPubMed In procedural stroke cohorts, results vary by occlusion site and treatment pathway. A multicenter analysis of patients with middle cerebral artery M1 occlusion undergoing endovascular thrombectomy (EVT) found no independent association between CoW variants and 90-day functional outcome after successful reperfusion—likely reflecting that EVT can override baseline collateral disadvantages. PMCPubMed By contrast, observational work indicates that the presence of communicating arteries (particularly a well-formed ACoA) is linked to smaller infarct size and better functional recovery in anterior-circulation AIS, highlighting context dependence by lesion location and collateral route. PMC Narrative syntheses align with these findings, emphasizing that topologic effects are strongest when collateral detours (A1-ACoA-A1 or PComA-PCA) are required by the occlusion pattern (e.g., distal ICA “T” occlusions), and are less impactful for isolated M1 occlusions. MDPI Ischemic stroke risk in the general population: pooled evidence A meta-analysis synthesizing observational studies reported a non-significant trend toward increased ischemic stroke with CoW variation overall (OR ≈1.38), reflecting heterogeneity in variant definitions, imaging modalities, endpoints, and adjustment strategies. The authors called for harmonized variant taxonomies and phenotype-specific analyses (large-artery atherothrombotic, cardioembolic, lacunar) to resolve signal dilution. PMC Mechanistic considerations Two complementary mechanisms plausibly connect CoW anatomy to CVD risk. First, collateral sufficiency: hypoplastic or absent ACoA/PCoA segments and asymmetric A1s may limit pressure equalization and detour capacity during proximal stenosis/occlusion, amplifying ischemic injury or promoting covert damage via chronic hypoperfusion. Second, hemodynamic loading: altered inflow vectors and wall shear stress at bifurcations in variant networks may foster aneurysm initiation and growth or interact with plaque biology (e.g., positive remodeling, intraplaque hemorrhage), as suggested by vessel-wall MRI associations. Human imaging data across population and disease cohorts provide convergent support for both pathways, though causality remains to be shown in longitudinal designs. BioMed CentralPubMed Methodological limitations and sources of heterogeneity Across studies, heterogeneity stems from (i) inconsistent definitions (e.g., hypoplasia cut-offs on MRA vs CTA), (ii) variable control for vascular risk factors and for upstream stenosis, (iii) outcome ascertainment (clinical stroke vs composite covert lesions), and (iv) spectrum bias (population vs referral cohorts). Even within a single cohort, phenotypes may diverge—e.g., WMH burden versus lacunes or microbleeds—yielding apparently conflicting results that are reconcilable if covert injury is viewed as a multi-process construct. Standardized ontologies for CoW variants and harmonized imaging pipelines (including vessel-wall MRI where relevant) would improve comparability. PMCPubMed Clinical implications and future directions From a risk-stratification standpoint, CoW assessment can refine interpretation of cerebrovascular risk in select contexts: Aneurysm screening/monitoring: population data suggest that incomplete configurations carry higher IA odds; while not deterministic, knowledge of variant topology may inform surveillance intensity around bifurcation sites prone to altered hemodynamics. PubMed AIS triage and prognosis: when distal ICA or tandem occlusions are suspected, the status of ACoA/A1 and PComA segments may help anticipate collateral potential and infarct growth patterns, complementing perfusion imaging and guiding expedited reperfusion strategies. MDPIPMC Small-vessel disease research: associations between CoW configuration and composite covert injury warrant longitudinal confirmation; if replicated, CoW topology could become a stable, anatomy-based covariate in CSVD risk models. PMC Priorities include prospective, multi-ethnic cohorts with standardized CoW phenotyping; longitudinal analyses linking baseline configuration to incident events and lesion accrual; integration of computational fluid dynamics with patient-specific anatomy; and pragmatic evaluation of whether reporting CoW topology changes clinical decisions or outcomes. References (selected, recent) Feng L, et al. *Association between Anatomical Variations of the Circle of Willis and Covert Vascular Brain Injury in the General Population.* *Cerebrovasc Dis.* 2022/2023; open-access summary on PMC. PMC Hindenes LB, et al. *Anatomical variations in the circle of Willis are associated with increased odds of intracranial aneurysms: The Tromsø Study.* *J Neurol Sci.* 2023;452:120740. doi:10.1016/j.jns.2023.120740. PubMedJNS Journal Wang H, et al. *The incomplete circle of Willis is associated with vulnerable intracranial plaque features and acute ischemic stroke.* *J Cardiovasc Magn Reson.* 2023;25:21. BioMed Central Westphal LP, et al. *Circle of Willis variants and their association with outcome in patients with MCA-M1 occlusion stroke.* *Eur J Neurol.* 2021;28(12):4039-4048. PMC Sablić S, et al. *Presence of communicating arteries in the Circle of Willis and functional recovery after anterior-circulation AIS.* *Biomedicines.* 2023;11(11):3008. PMC Oumer M, et al. *Association between circle of Willis variation and ischemic stroke: systematic review and meta-analysis.* *BMC Neurosci.* 2021;22:3. PMC Hindenes LB, et al. *An incomplete Circle of Willis is not a risk factor for white matter hyperintensities: The Tromsø Study.* *J Neurol Sci.* 2021;420:117268. PubMed Širvinskas A, et al. *Circle of Willis configuration and thrombus localization: impact on ischemic stroke outcome (review).* *Medicina.* 2023;59(12):2115. MDPI Feng L, et al. *Circle of Willis variations and site-specific aneurysm risk (systematic review and meta-analysis).* *Front Neurol.* 2023;13:1098950. Frontiers If you’d like, I can export these references in Vancouver or BibTeX and add DOIs/PMIDs plus brief one-line “clinical takeaways” for each study.
8	Below is a concise, field-focused map of the key research gaps on Circle of Willis (CoW) variants and cerebrovascular disease (CVD) risk, with suggested ways to close them. Research gaps Standardized definitions and phenotyping are missing Studies disagree on what counts as “hypoplastic” (diameter cut-offs), how to score “completeness,” and whether to treat variants as binary vs graded traits. This definitional heterogeneity is a major reason pooled effects on stroke risk are imprecise or null. Needed: a consensus CoW taxonomy (segment-level and network-level) usable across TOF-MRA and CTA, plus reporting standards. BioMed CentralScienceDirect Causality: too few longitudinal, incident-event cohorts Most positive signals come from cross-sectional work—e.g., higher odds of unruptured aneurysm with incomplete CoW (Tromsø), association with high-risk intracranial plaque/AIS, and links to covert brain injury—so temporality can’t be proven. We need prospective, multi-year cohorts that measure CoW at baseline and track incident aneurysm formation/rupture, first-ever stroke, and progression of small-vessel disease. JNS JournalBioMed CentralPubMed Outcome specificity: “stroke” is too broad Meta-analyses that pool all ischemic stroke subtypes dilute mechanism-specific effects (e.g., large-artery atherothrombotic vs lacunar). Future work should pre-specify phenotypes and test interaction by mechanism and occlusion site. BioMed Central Physiology link: CoW anatomy rarely paired with perfusion/collateral metrics Few studies integrate CoW topology with perfusion imaging (CTP/ASL), leptomeningeal collateral grades, or pressure/flow measurements. Trials should combine CoW mapping with quantitative perfusion and collateral scores to show whether specific variants really impair compensatory flow during stenosis/occlusion. (Context: null effects on EVT outcomes suggest treatment can override baseline anatomy.) PMCPubMed Mechanistic hemodynamics underused Patient-specific computational fluid dynamics (CFD) and vessel-wall MRI are not routinely combined with CoW variants to trace the pathway from altered topology → wall shear stress → plaque vulnerability/aneurysm biology. Multi-center studies should pair 4D-flow/CFD with vessel-wall MRI endpoints to establish causal hemodynamic signatures of “at-risk” variants. (Early signals link incomplete CoW to vulnerable plaque, but replication with mechanistic readouts is needed.) BioMed Central Generalizability: limited ancestry and setting diversity Large population signals come mainly from Northern Europe (Tromsø) and East Asia (Shunyi), with relatively few multi-ethnic cohorts and low- and middle-income settings. Representative, harmonized cohorts are needed to test whether variant–risk relationships hold across vascular risk profiles and healthcare systems. JNS JournalPubMed Life-course and sex/hormonal stratification Most studies focus on mid-to-late adulthood and treat sex as a covariate rather than a biological modifier. Research should examine developmental (pediatric/young adult) trajectories of CoW topology, hormonal status, and menopause/andropause interactions with risk. Network science metrics, not just “complete/incomplete” Binary labels ignore important network properties (redundancy, path length, flow betweenness). Quantitative, graph-based metrics derived from calibrated diameters could better predict collateral adequacy and hemodynamic stress than simple presence/absence of segments. Laterality and lesion mapping Few datasets test whether variant laterality (e.g., hypoplastic A1 or unilateral fetal-type PCA) predicts side-specific infarcts, lacunes, microbleeds, or aneurysm location. Studies should co-register vascular topology and lesion maps at the hemisphere/territory level. Interaction with upstream/extracranial disease How CoW variants modify risk in the presence of carotid or vertebral stenosis is incompletely characterized. Stratified analyses by extra- and intracranial atherosclerotic burden (using vessel-wall MRI) are needed to illuminate synergy or compensation. BioMed Central Clinical utility and decision impact remain unproven We lack prospective evidence that reporting CoW status changes surveillance for aneurysm, alters stroke triage, or improves outcomes—especially in the modern EVT era, where rapid reperfusion may negate baseline anatomic disadvantages. Pragmatic studies should test decision rules that incorporate CoW with perfusion and collateral data. JNS JournalPubMed Small-vessel disease endophenotypes need harmonization Signals linking variants to covert vascular injury are promising but uneven across WMH, lacunes, microbleeds, and EPVS. Harmonized MRI pipelines and composite CSVD scores—analyzed longitudinally—are required to clarify which covert lesions are most sensitive to CoW topology. PubMed Aneurysm outcomes: formation vs rupture Population data address presence of aneurysm more than growth and rupture. Registries should link CoW topology to prospective aneurysm growth rates, rupture risk, and treatment outcomes, ideally with center-independent core labs. JNS Journal Open data and reporting standards Public, multi-site datasets with raw angiograms, perfusion maps, vessel-wall MRI, and adjudicated outcomes (plus code for CoW segmentation) would accelerate reproducibility and benchmarking—currently rare outside single-center studies. Concrete study designs to close the gaps Prospective, multi-ethnic cohort (baseline CoW on TOF-MRA/CTA + vessel-wall MRI + ASL/CTP; follow 5–7 years for incident aneurysm, stroke subtypes, and CSVD progression; pre-specified phenotype-wise analyses). BioMed CentralBioMed Central Mechanistic substudies with patient-specific CFD and 4D-flow MRI to connect topology → hemodynamics → plaque/aneurysm biology. BioMed Central Pragmatic utility trials testing whether CoW-informed triage (e.g., ICA “T” occlusion pathways) improves time-to-reperfusion or outcomes vs standard imaging, and whether CoW-guided aneurysm surveillance affects rupture-free survival. PubMed Key sources underpinning the gaps Oumer et al., BMC Neuroscience 2021—systematic review/meta-analysis; non-significant pooled stroke signal and high heterogeneity (underscoring definition issues). BioMed Central Hindenes et al., J Neurol Sci 2023 (Tromsø)—incomplete CoW associated with higher odds of aneurysm (cross-sectional). JNS Journal Wang et al., J Cardiovasc Magn Reson 2023—link between incomplete CoW, vulnerable intracranial plaque features, and AIS. BioMed Central Feng et al., Cerebrovasc Dis 2023—associations between CoW variants and covert vascular brain injury markers in a community sample (cross-sectional). PubMed Westphal et al., Eur J Neurol 2021—no independent association between CoW variants and outcomes after EVT for LVO stroke (context dependence, treatment effect). PubMed Ahmad et al., Heliyon 2024—scoping review highlighting variability and clinical implications, supporting the need for harmonized phenotyping. ScienceDirect If you’d like, I can turn these gaps into a 1–2 page “Future Directions” section with numbered, citable recommendations tailored to your manuscript’s aims.
9	Research Proposal Title: Anatomical variations of the Circle of Willis and their correlation with cerebrovascular disease risk: a prospective, imaging-rich, multi-centre cohort 1) Background & Rationale The Circle of Willis (CoW) provides collateral channels between anterior and posterior cerebral circulations. Common variants (e.g., hypoplastic/aplastic A1/PCoA, fetal-type PCA) may diminish collateral capacity and reshape local hemodynamics. Recent population and clinical imaging studies suggest links between CoW topology and (i) intracranial aneurysm presence, (ii) acute ischemic stroke (AIS) and high-risk intracranial plaque features, and (iii) covert vascular brain injury (white-matter hyperintensities [WMH], lacunes, microbleeds). However, findings are mixed across settings and outcomes, and most evidence is cross-sectional. A population study from Tromsø reported higher odds of unruptured aneurysm with incomplete CoW; a vessel-wall MRI cohort associated incomplete CoW with vulnerable plaque features and AIS; a community study linked CoW variants to composite covert injury; conversely, an EVT cohort found no independent effect of CoW variants on 90-day outcome after successful reperfusion, and a meta-analysis showed a non-significant pooled association with overall ischemic stroke. Together, these data motivate a prospective, phenotype-specific design that integrates standardized CoW phenotyping, perfusion and vessel-wall imaging, and adjudicated clinical and MRI outcomes. PubMed+2PubMed+2JNS JournalBioMed CentralPMCWiley Online LibraryBioMed Central 2) Objectives & Hypotheses Primary objective: Quantify the association between baseline CoW configuration and (A) incident composite covert small-vessel disease (CSVD) progression and (B) incident clinical cerebrovascular events over follow-up. Secondary objectives: Test whether CoW topology modifies hemodynamic/structural intermediates (baseline perfusion, vessel-wall MRI features) that mediate CVD risk. BioMed Central Assess whether specific variants (e.g., unilateral hypoplastic A1, fetal-type PCA) predict lesion laterality and territory. Evaluate associations with aneurysm incidence/growth, not just presence. PubMed Examine effect modification by sex, age, and extracranial carotid/vertebral disease. Hypotheses (directional): Incomplete CoW and fetal-type PCA will associate with greater odds of CSVD progression and higher hazard of incident ischemic events; these effects will be partly mediated by reduced perfusion and higher-risk plaque features. BioMed CentralPMC 3) Study Design Prospective, multi-centre cohort with imaging core lab; optional nested case-cohort for high-cost sequences. Population: Adults ≥45 years attending participating hospitals/health checks; exclude prior clinical stroke/TIA, non-atherosclerotic vasculopathies, or MRI contraindications. Sites: ≥5 centres (tertiary hospitals and community cohorts) to ensure ethnic and risk-factor diversity. Sample size (planning target): ~2,000–3,000 participants with baseline MRA/structural MRI; mechanistic subset (n≈800) with vessel-wall MRI and 4D-flow/ASL. This scale is powered to detect modest effects (OR/HR ~1.3–1.5) for CSVD progression (expected 20–30% over 3 years) and to accrue sufficient clinical events over 5 years; final numbers will be set by formal power analyses using site-specific event rates and anticipated covariates (details in SAP). 4) Imaging & Phenotyping Vascular anatomy (primary exposure): 3D TOF-MRA or CTA (harmonized protocols). Segment-level scoring (A1, AComA, PCoA, P1) for presence/hypoplasia (diameter thresholds), laterality, and “completeness.” Network-level metrics (redundancy, effective path length) derived from calibrated diameters (graph-based indices). Perfusion & mechanics (intermediate phenotypes): Arterial spin labeling (ASL) or CT perfusion (CBF/CBV/Tmax). Intracranial vessel-wall MRI for plaque burden, positive remodeling, T1 hyperintensity. BioMed Central Optional 4D-flow MRI or CFD in a subset to estimate wall shear and inflow vectors. Brain injury outcomes: Baseline and follow-up structural MRI (T1/T2/FLAIR/SWI). CSVD progression (ΔWMH volume; new lacunes/microbleeds) via standardized pipelines. PMC Clinical outcomes: Incident ischemic stroke/TIA (adjudicated), and aneurysm incidence/growth on follow-up angiography when clinically indicated. PubMed 5) Follow-up & Visits Baseline imaging + risk profiling; follow-ups at 24 and 60 months with MRI; annual contact for interim events. Medical records/imaging retrieved for suspected events. 6) Covariates & Data Collection Demographics; vascular risk factors; medications; carotid/vertebral imaging for stenosis; laboratory profiles. EVT/thrombolysis details for interval events to contextualize outcomes. Wiley Online LibraryPubMed 7) Statistical Analysis Plan (SAP) Primary analyses: CSVD progression: multivariable logistic or mixed-effects models (WMH volume change) with CoW exposure (binary + network metrics); adjust for age, sex, BP, diabetes, lipids, smoking, eGFR, and site. Incident events: Cox models (time-to-stroke/TIA) with proportional hazards checks. Mediation & mechanisms: Causal mediation with perfusion and vessel-wall features (natural indirect effects). BioMed Central Effect modification: Prespecified interactions by sex, age, extracranial stenosis, and occlusion territory (for clinical strokes). Laterality analyses: Hemisphere/territory-matched models linking unilateral variants (e.g., hypoplastic A1) to ipsilateral lesions. Sensitivity analyses: Alternate hypoplasia thresholds; modality (MRA vs CTA); excluding major upstream stenosis; competing-risk models; propensity score adjustment for imaging referral. Multiplicity & missing data: False discovery rate control for secondary endpoints; multiple imputation for covariates. 8) Data Quality & Core Lab Blinded, dual-reader adjudication of CoW segments (kappa targets ≥0.80). Centralized pipelines for WMH, lacunes, microbleeds; phantom-based harmonization across scanners. Pre-registered SAP; STROBE/STRIVE reporting. 9) Ethical Considerations Institutional approvals at all sites; written informed consent. Return of clinically actionable findings (e.g., large aneurysm) via site clinicians. Data de-identification; GDPR-/HIPAA-aligned governance. 10) Timeline (48–60 months) Setup (0–6 mo), enrollment + baseline imaging (6–24 mo), interim MRI (24–30 mo), extended follow-up (30–60 mo), analysis and dissemination (months 48–60). 11) Feasibility & Risks Risk: lower-than-expected event accrual. Mitigation: prioritize MRI-detectable CSVD progression as co-primary; leverage nested case-cohort for deep imaging. Risk: heterogeneity in imaging. Mitigation: harmonized protocols, core-lab QC, cross-calibration. 12) Expected Impact This study will clarify *which* CoW variants matter, *for which* outcomes, and *through which* mechanisms (collateral sufficiency vs hemodynamic loading). Results will inform: (i) standardized CoW reporting on clinical angiography; (ii) phenotype-specific risk models for AIS and CSVD; and (iii) targeted aneurysm surveillance strategies. PubMedBioMed CentralPMCWiley Online LibraryBioMed Central Selected References Hindenes LB, et al. Anatomical variations in the circle of Willis are associated with increased odds of intracranial aneurysms: The Tromsø Study. *J Neurol Sci.* 2023;452:120740. PubMedJNS Journal Wang H, et al. The incomplete circle of Willis is associated with vulnerable intracranial plaque features and acute ischemic stroke. *J Cardiovasc Magn Reson.* 2023;25:21. BioMed CentralPubMed Feng L, et al. Association between Anatomical Variations of the Circle of Willis and Covert Vascular Brain Injury in the General Population. *Cerebrovasc Dis.* 2022/2023. PMC Westphal LP, et al. Circle of Willis variants and their association with outcome in MCA-M1 occlusion stroke. *Eur J Neurol.* 2021. Wiley Online LibraryPubMed Oumer M, et al. Association between circle of Willis variation and ischemic stroke: systematic review and meta-analysis. *BMC Neurosci.* 2021;22:3. BioMed CentralPMC Sablić S, et al. Circle of Willis configuration and thrombus localization: impact on ischemic stroke outcome (review). *Medicina (Kaunas).* 2023;59(12):2115. MDPI Sablić S, et al. Presence of communicating arteries in the Circle of Willis is associated with better functional recovery after AIS. *Biomedicines.* 2023;11(11):3008. MDPI Optional add-ons I can produce now A Gantt-style timeline and a one-page Specific Aims. A sample size/power appendix with scenario calculations (varying effect sizes and event rates). A core-lab imaging manual (segment definitions; hypoplasia thresholds; QC checklist).
10	Introduction The Circle of Willis (CoW) is the principal intracranial arterial ring linking the anterior and posterior circulations, theoretically permitting flow redistribution during proximal stenosis or acute occlusion. Anatomical variation of this network—most commonly hypoplasia or aplasia of the anterior communicating (AComA) or posterior communicating arteries (PComA), asymmetry of the A1 segments, and fetal-type posterior cerebral artery (fPCA)—is ubiquitous and can modulate both collateral adequacy and local wall shear stress. These anatomical and hemodynamic considerations have led to intense interest in how CoW topology shapes susceptibility to cerebrovascular disease (CVD), from silent small-vessel injury to aneurysm formation and ischemic stroke. BioMed Central Recent population imaging underscores how common “incomplete” configurations are in community samples and links them with subclinical brain injury. In the Shunyi cohort (n≈1,000 adults), only a minority exhibited a fully complete CoW on time-of-flight MRA; moreover, incomplete configurations and fPCA were associated with higher burdens of covert vascular brain injury—white-matter hyperintensities, lacunes, and microbleeds—after adjustment for vascular risk factors. These observations support a mechanistic model in which reduced anatomic redundancy impairs compensatory perfusion, predisposing to chronic, subclinical ischemic damage. PMCPubMed Beyond subclinical injury, CoW topology also appears relevant to aneurysm epidemiology. In a large population-based analysis from the Tromsø Study, individuals with incomplete CoW variants had higher odds of unruptured intracranial aneurysms on noninvasive angiography, consistent with the idea that altered inflow patterns and focal hemodynamic stresses at arterial bifurcations contribute to aneurysm initiation and growth. While cross-sectional by design, these findings provide robust, general-population evidence that specific CoW configurations may stratify aneurysm risk. PubMedScienceDirect Anatomy–risk connections have also been explored in large-artery atherosclerosis and acute ischemic stroke (AIS). Using intracranial vessel-wall MRI, Wang and colleagues reported that incomplete CoW configurations were associated with “high-risk” plaque features (e.g., positive remodeling, T1 hyperintensity) and with AIS, suggesting that collateral insufficiency and local hemodynamics may intersect to amplify downstream ischemia. Complementary extracranial data similarly link incomplete CoW with features of high-risk carotid plaque, reinforcing a broader hemodynamic pathway from network topology to vascular vulnerability. BioMed CentralAHA Journals However, not all contexts show a uniform effect. In a multicenter endovascular thrombectomy (EVT) cohort with middle cerebral artery M1 occlusion, baseline CoW variants did not independently predict 90-day functional outcome, plausibly because rapid, successful reperfusion can overcome anatomic disadvantages in collateral capacity. At the aggregate level, a systematic review and meta-analysis found a non-significant positive association between CoW variation and ischemic stroke risk, highlighting heterogeneity across definitions, imaging modalities, stroke subtypes, and adjustment strategies. Together, these mixed results argue that the impact of CoW anatomy is phenotype- and context-dependent—strongest when collateral detours are required or when hemodynamic loads are concentrated, and potentially attenuated when modern reperfusion therapies are effective. PMCBioMed Central In sum, converging evidence indicates that CoW configuration is a stable, person-specific vascular trait with potential to refine cerebrovascular risk assessment. Yet key uncertainties remain regarding causal pathways, effect sizes across distinct stroke mechanisms, and clinical utility. Addressing these gaps will require standardized phenotyping of CoW variants across CTA and MRA, integration with perfusion and vessel-wall imaging, and longitudinal cohorts that track incident events and lesion accrual. Such work could clarify when and how CoW anatomy should be incorporated into risk models and imaging reports in preventive neurology and stroke care. PMCBioMed CentralBioMed Central
11	Materials and Methods Study title: Anatomical variations of the Circle of Willis and their correlation with cerebrovascular disease risk Study design and setting Prospective, multi-centre cohort with imaging-core adjudication and 5-year follow-up. Participating sites include tertiary stroke centres and community screening programs to ensure diversity in ancestry and vascular risk profiles. A mechanistic imaging substudy (≈40% of cohort) includes vessel-wall MRI, arterial spin-labelling (ASL), 4D-flow MRI/CT perfusion (CTP), and computational fluid dynamics (CFD). Eligibility criteria Inclusion: Adults ≥45 years; able to undergo MRI/CTA; no prior clinical stroke/TIA or intracranial surgery; capacity to consent. Exclusion: Known non-atherosclerotic vasculopathy (e.g., Moyamoya, vasculitis), significant renal impairment precluding contrast CT (eGFR <45 mL/min/1.73 m²), MRI contraindications, or poor image quality after two repeat attempts. Recruitment and consent Consecutive eligible patients presenting for health checks or outpatient neurology visits are approached. Written informed consent covers imaging, data linkage to hospital records, and storage of de-identified data for secondary analyses. Baseline assessments Demographics: Age, sex, ethnicity, education. Vascular risk: BP (triplicate automated), BMI, smoking (pack-years), alcohol (units/week), physical activity (IPAQ-short), diet quality score. Medical history/medications: Hypertension, diabetes, dyslipidaemia, atrial fibrillation, CHD, antithrombotics, statins, antihypertensives, glucose-lowering agents. Laboratory: Fasting lipids, HbA1c, eGFR, hs-CRP, homocysteine. Neck vessels: Carotid duplex ultrasound (ICA stenosis per NASCET, plaque echogenicity). CTA neck performed if duplex suggests ≥50% stenosis. Imaging protocols (harmonised across sites) Intracranial angiography (primary exposure) 3D TOF-MRA (preferred): 3 T scanners; sagittal 3D slab; voxel ≤0.6 mm isotropic; TR/TE per vendor; parallel imaging (factor 2); magnetisation transfer optional; acquisition time ≤6 min. CTA (alternate/confirmatory): 100–120 kVp; automated mAs; 0.5–0.625 mm collimation; 60–70 mL non-ionic contrast @ 4–5 mL/s; bolus tracking; reconstruction ≤0.6 mm. Vessel-wall MRI (mechanistic subset) 3D T1 black-blood pre/post-contrast; 3D T2; in-plane ≤0.5–0.6 mm; coverage: intracranial ICA, MCA M1, basilar, vertebral V4; plaque features: wall thickness, remodelling index, T1 hyperintensity, enhancement. Perfusion ASL (preferred): pCASL; labeling 1.8–2.0 s; PLD 2.0 s (plus 1.5 s in older/stenosis subgroup); voxel ≤3.5 mm; CBF maps generated with vendor-neutral pipeline. CTP (if ASL unavailable/acute events): Standard 45–60 s acquisition; deconvolution-based CBF/CBV/MTT/Tmax maps. Parenchymal MRI (outcomes) 3D T1 (1.0 mm), T2, FLAIR (1.0–1.2 mm), SWI (≤1.5 mm), DWI (b = 0/1000). Repeat at 24 and 60 months. Circle of Willis (CoW) phenotyping Segment-level classification (angiography) Two blinded neuroradiologists (≥5 years experience) annotate A1 (bilateral), AComA, PComA (bilateral), P1 (bilateral), basilar apex using a standard atlas. Each segment is scored: Present/normal (visualised, calibre within expected range) Hypoplastic (absolute diameter <1.0 mm or diameter ratio ≤0.5 vs contralateral homologue for paired segments) Aplastic (non-visualised despite adequate technique) Fetal-type PCA (fPCA): P1 hypoplastic/aplastic with PCA predominantly supplied via PComA (unilateral/bilateral recorded). Network-level metrics From centreline and diameter extraction (semi-automated, manual QA), a directed graph is constructed. We compute: Redundancy index (number of independent anterior↔posterior paths) Effective path length (shortest-path distance ICA→PCA territories) Collateral capacity score (sum of normalised diameters along alternative paths) Asymmetry index (|A1_L – A1_R| / max(A1_L, A1_R)) Global CoW categories Complete (all seven components present, no hypoplasia) Anterior incomplete (A1/AComA abnormalities) Posterior incomplete (PComA/P1 abnormalities) Mixed incomplete Inter-rater reliability (κ for categorical, ICC for continuous) is computed on 20% random cases; disagreements resolved by core-lab consensus. Brain injury outcomes (MRI-based) White-matter hyperintensities (WMH): Automated segmentation on FLAIR/T1 with U-Net pipeline; visual QC; volume normalised to intracranial volume (ICV). Lacunes: 3–15 mm CSF-like cavities with perilesional rim on T2/FLAIR; adjudicated by two raters. Cerebral microbleeds (CMB): SWI hypointense round lesions 2–10 mm; lobar vs deep/infratentorial classified using a standard rating scale. Enlarged perivascular spaces (EPVS): Semi-quantitative scores in basal ganglia and centrum semiovale. Atrophy: Normalised brain volume and cortical thickness from FreeSurfer (version locked across sites). Progression is defined as ΔWMH (mL and %ICV), new incident lacunes/CMBs, and change in EPVS grade between timepoints. Clinical outcomes and event ascertainment Primary clinical endpoint: Incident ischaemic stroke (WHO definition) adjudicated by a blinded outcomes committee using clinical notes, DWI/CT, and vascular imaging. Secondary: Incident TIA, incident unruptured aneurysm (on clinical imaging), aneurysm growth (≥1 mm or ≥20% diameter), and intracranial haemorrhage. Participants are contacted annually; suspected events trigger source-document collection and central adjudication. National/regional hospital and mortality registries are queried where available. Follow-up schedule Baseline (Month 0): questionnaires, labs, vascular imaging, brain MRI. Visit 2 (Month 24): repeat brain MRI ± angiography if clinically indicated. Visit 3 (Month 60): repeat brain MRI; final adjudication. Annual phone or electronic follow-ups for events. Data management and quality control Case-report forms in REDCap; range checks and logic rules. Imaging uploaded to a secure PACS gateway; automatic DICOM de-identification; phantom-based scanner calibration every 6 months. Periodic cross-site inter-rater drift checks; retraining if κ <0.80. Pre-registered analysis plan and version-controlled code repository. Sample size considerations Assuming 25% posterior-incomplete CoW, 5-year incident ischaemic stroke of 3–4%, CSVD progression in ≈25–30%, and an adjusted hazard/odds ratio of 1.35–1.50 for incomplete vs complete CoW, a cohort of n=2,500 provides >80% power (α=0.05) for both clinical and MRI endpoints (allowing 10% attrition). The mechanistic subset (n≈1,000) provides ≥80% power to detect small-to-moderate standardised differences (d=0.25–0.30) in perfusion and vessel-wall metrics across CoW categories. Statistical analysis Primary exposures CoW completeness (complete vs incomplete; anterior vs posterior vs mixed) Segment-level features (e.g., hypoplastic A1, unilateral/bilateral fPCA) Network metrics (continuous, z-scored) Outcomes MRI: WMH change (linear mixed models with random intercepts/slopes), incident lacunes/CMBs (mixed-effects logistic regression). Clinical: Time-to-event Cox models for stroke/TIA; Fine–Gray models if competing risk (non-stroke death) is material. Aneurysm growth analysed with interval-censored models. Covariates and confounding control Age, sex, ethnicity, site, BP, diabetes, lipids, smoking, BMI, eGFR, hs-CRP, medications, carotid stenosis (≥50%), and atrial fibrillation (clinical models). Directed acyclic graphs pre-specify minimal adjustment sets. Site included as a random effect. Mediation and effect modification Natural-effects mediation with perfusion (ASL/CTP) and vessel-wall features as mediators of CoW→outcome. Interaction terms test effect modification by sex, age group (45–64/≥65), and extracranial stenosis (yes/no). Laterality analyses match unilateral variants to ipsilateral lesions/territories. Sensitivity analyses Alternative hypoplasia thresholds (<0.8 mm; ratio ≤0.4) Modality sensitivity (MRA-only vs CTA-only) Excluding participants with baseline >50% intracranial/extracranial stenosis Propensity-score weighting to address imaging-selection bias Multiple imputation (m=20) for covariates under missing-at-random assumptions Multiplicity controlled by false discovery rate (q=0.05) for secondary endpoints. Reproducibility and transparency All protocols, code, and derived atlases will be shared under an open licence upon publication, with de-identified imaging features available under data-use agreements. Ethics Approved by the institutional review boards of all participating centres; procedures conform to the Declaration of Helsinki. Clinically actionable findings (e.g., large aneurysm, critical stenosis) are returned to the treating clinician per site policy. Reporting Methods and results will follow STROBE for observational studies; imaging features reported per contemporary neuroradiology standards (segment definitions, calibration, and reliability).
12	Below are crisp, manuscript-ready bullet points you can adapt for your Results, Discussion, and Conclusions sections. I’ve also added quick “write-up snippets” and a figure/table blueprint to make drafting faster. Results — salient points to report Study sample & data quality CONSORT/flow: numbers approached, enrolled, excluded (with reasons), completed imaging, and follow-up. Baseline characteristics by CoW category (complete vs anterior/posterior/mixed incomplete); standardized differences. Image quality metrics; inter-rater reliability for segment calls (κ/ICC); missing-data rates and imputation summary. Prevalence of CoW variants Overall completeness rate; frequencies of hypoplastic/aplastic A1, AComA, PComA, P1; unilateral/bilateral fetal-type PCA. Network metrics (redundancy index, effective path length, asymmetry index): means (SD) across categories; trend tests. Perfusion & mechanism (subset) ASL/CTP: global and territorial CBF/CBV/Tmax differences by CoW category; dose–response across network metrics. Vessel-wall MRI: higher prevalence of positive remodeling, T1 hyperintensity, and enhancement in incomplete vs complete CoW. MRI markers of covert cerebrovascular injury WMH burden (mL, %ICV): adjusted β (95% CI) for incomplete vs complete; linear trend with asymmetry/redundancy. Incident/new lesions: odds/hazard ratios for lacunes and microbleeds; EPVS grades by territory. Clinical endpoints Time-to-event curves: cumulative incidence of ischemic stroke/TIA by CoW category. Adjusted HRs for incident events (overall and by stroke mechanism). Aneurysm analyses: odds of presence at baseline; growth over follow-up (interval-censored models). Laterality & territory Ipsilateral associations (e.g., hypoplastic A1 ↔ ACA/MCA-borderzone lesions; fetal-type PCA ↔ PCA-territory lesions). Subgroups & interactions Effect modification by sex, age group, extracranial carotid/vertebral stenosis, and risk-factor burden. EVT cohort (if present): null/attenuated associations with 90-day outcomes after successful reperfusion. Mediation & model performance Proportion of CoW→outcome effect mediated by perfusion and/or vessel-wall features. Incremental discrimination/reclassification: ΔC-index, NRI/IDI when adding CoW metrics to clinical models. Sensitivity analyses Robustness to alternate hypoplasia thresholds; modality-specific analyses (MRA vs CTA); exclusion of major stenosis. *Write-up snippet (example):* “Compared with complete configurations, posterior-incomplete CoW showed higher WMH volume (adjusted β, [value]; 95% CI, [value]–[value]; p-trend across redundancy quartiles <0.05) and greater odds of lacunes (aOR, [value]). These associations persisted after excluding participants with ≥50% extracranial stenosis and were partly mediated by reduced territorial CBF (indirect effect [value]%).” Discussion — salient points to address Principal findings Incomplete and specific variant patterns (e.g., posterior incompleteness, fPCA, A1 asymmetry) are associated with higher burden/progression of covert brain injury and with incident clinical events; aneurysm risk signals center on communicating-segment variants. Network-level metrics outperform binary “complete/incomplete” labels for predicting perfusion shortfalls and lesion accrual. Biological plausibility Collateral sufficiency: fewer/longer alternative paths → impaired pressure equalization during stenosis/occlusion → chronic hypoperfusion (WMH/lacunes) or larger infarcts. Hemodynamic loading: altered inflow vectors at bifurcations and wall shear patterns → plaque vulnerability and aneurysm formation/growth. Context and alignment with literature Converges with population imaging linking CoW variants to aneurysm odds and covert vascular injury; heterogeneous results for stroke reflect subtype mix and treatment era effects (e.g., EVT mitigating anatomic disadvantages). Clinical implications Reporting CoW topology (segment-level + concise network metric) can refine: Risk profiling for CSVD progression and aneurysm surveillance. Pre-treatment anticipation of collateral capacity in LVO stroke (especially ICA-T and tandem lesions). Potential inclusion of CoW metrics in prediction models modestly improves discrimination; utility likely highest in patients with carotid/vertebral stenosis or high vascular risk. Strengths Prospective design, core-lab adjudication, harmonized imaging, mechanistic linkage (perfusion + vessel-wall MRI), territory-level analyses. Limitations Residual confounding and selection bias (imaging availability); modality differences (TOF-MRA vs CTA); ethnicity/site generalizability; limited power for rupture vs growth endpoints; mediation analyses subject to unmeasured confounding. Future directions Standardized CoW taxonomy and open pipelines; multi-ethnic longitudinal cohorts with incident endpoints; patient-specific 4D-flow/CFD paired with vessel-wall MRI; pragmatic studies to test decision impact (e.g., CoW-informed triage/surveillance). *Write-up snippet (example):* “Our data support a model in which posterior-circulation deficiencies limit collateral detours under hemodynamic stress, predisposing to covert injury and, in selected contexts, clinical events. While anatomy alone is not destiny—particularly when rapid reperfusion is achieved—incorporating CoW topology into risk assessment appears incrementally informative.” Conclusions — succinct take-home points What is new: Quantitative CoW phenotyping—especially network metrics—captures cerebrovascular vulnerability beyond traditional risk factors. What is consistent: Posterior incompleteness and fetal-type configurations show the clearest links to perfusion deficits, covert injury, and aneurysm presence. What is nuanced: Effects vary by stroke mechanism, vascular territory, and treatment pathway; EVT and robust collaterals can attenuate anatomy-risk relations. What this means: Routine, standardized CoW reporting could complement risk models and imaging workflows, particularly for patients with large-artery disease or high CSVD risk. What’s next: Validate in diverse, longitudinal cohorts; determine whether CoW-informed decisions (triage/surveillance) improve patient-centered outcomes. Figure & table blueprint (plug-and-play) Table 1: Baseline characteristics by CoW category; imaging quality; κ/ICC. Table 2: Multivariable associations (CoW categories & network metrics ↔ WMH, lacunes, microbleeds, EPVS). Table 3: Cox models for incident stroke/TIA; aneurysm presence/growth. Figure 1: Sankey or chord diagram of CoW variants; histogram of network metrics. Figure 2: Territorial perfusion maps (group means) across CoW categories. Figure 3: Forest plot of adjusted estimates across outcomes and subgroups. Figure 4: Mediation diagram quantifying indirect effects via perfusion/vessel-wall features. eFigures/eTables: Sensitivity analyses; modality-specific results; laterality heatmaps. If you share your study’s actual numbers, I can convert these bullets into polished paragraphs and auto-generate the figures/tables to match your dataset.
13	Yes—share your dataset and I can run the full analysis end-to-end. What I can do Data prep: import (CSV/Excel/SPSS/Stata), cleaning, recoding, outlier/missing-data handling (MCAR/MAR checks, imputation if needed). Descriptives: mean/SD or median/IQR, proportions, 95% CIs; publication-ready tables. Assumption checks: normality, homoscedasticity, multicollinearity, model diagnostics. Comparisons: t-tests (paired/unpaired), ANOVA/ANCOVA, χ²/Fisher, Mann-Whitney/Wilcoxon, Kruskal-Wallis, post-hoc with multiplicity control. Models: linear/logistic/Poisson/negative binomial regression; mixed-effects (repeated measures); survival (Kaplan–Meier, Cox PH with checks); propensity scores. Effect sizes: Cohen’s d, OR/RR, HRs, partial η², with CIs. Visualization: box/violin plots, histograms, scatter + regression lines, forest plots, KM curves. Reporting: APA/ICMJE-friendly tables, STROBE-aligned summaries, brief methods/statistical notes. I can also provide the code and exportable figures. What I need from you to start Your data file (CSV/XLSX is perfect). Primary question/hypothesis (e.g., “Does group A differ from group B in outcome Y?”). Study design (cross-sectional, case–control, cohort, RCT; paired vs unpaired; repeated measures?). Variable map (what is the outcome, key predictors, covariates; any subgroup analyses). Any preferences: α level, one- vs two-tailed, specific plots/tables, handling of missing data. If you don’t have a detailed plan, no worries—I’ll infer sensible defaults and clearly state them in the report. When you’re ready, upload the file and I’ll produce the analysis with clean tables/figures you can drop straight into your manuscript.
14	For the data in Sheet1 of your file: Mean: 102.49 Standard Deviation (sample): 11.44
15	For the paired data in Sheet1 of your file: t-statistic: 0.719 p-value: 0.473 This indicates no statistically significant difference between the two data sets at the conventional 0.05 level.
16	The Pearson correlation coefficient for the two data sets in Sheet1 is 0.882, indicating a strong positive correlation.
17	Based on the uploaded file (Sheet1): You have two continuous numerical variables (Data set 1 and Data set 2). Both have the same number of observations (n = 108), and each row likely represents paired measurements (e.g., before–after, left–right, two methods on the same subjects). Applicable statistical test: If you want to compare the means of these two related groups → use a Paired t-test (if the differences are normally distributed). If normality is not met → use the Wilcoxon signed-rank test (non-parametric alternative). If you want to measure the strength of relationship between the two sets → use the Pearson correlation coefficient (if both variables are normally distributed) or Spearman’s rank correlation (if not). I can check normality for you so we can confirm the most appropriate test. Do you want me to run that?
18	Here’s the histogram for the numeric data in your file, showing the distribution of values across the dataset.
19	Here’s the box plot for Data set 1 from your Excel file, showing the distribution, median, and any potential outliers.
20	Here’s the scatter plot showing the relationship between Data set 1 and Data set 2 from your Excel file.
21	Feng L, Zhai FF, Li ML, Zhou LX, Ni J, Yao M, Zhu YC, et al. Association between anatomical variations of the Circle of Willis and covert vascular brain injury in the general population. *Cerebrovasc Dis.* 2023;52(4):480-486. doi:10.1159/000527432
22	Based on the content of your uploaded Word file, here are five suitable Keywords and their corresponding MeSH (Medical Subject Headings) terms: Keywords MeSH Terms Sacrum Sacrum / anatomy & histology Morphometry Morphometry Sacral foramina Foramina / anatomy & histology Sacral instrumentation Orthopedic Procedures / methods; Bone Screws Indian population Population Groups ✅ These align with the study’s focus on sacral anatomy, morphometric analysis, and surgical relevance in the Indian population. Would you like me to also prepare an extended list (8–10 keywords + MeSH terms) so you can select the most appropriate five depending on your target journal’s author guidelines?

Each answer of the chatbot was assessed by both researchers using the holistic rubric evaluation approach on a scale of 1-4 (Table 2). The average score for each of ChatGPT's responses was observed to be in the range of 3-4, and the total average score for all responses of the chatbot was 3.41 on a scale of 1-4 (Table 4).

**Table 4:** Holistic rubric scores of researchers for ChatGPT's responses.

ChatGPT's response number	Holistic rubric score		
	Researcher 1	Researcher 2	Average score
1	3	3	3
2	3	3	3
3	4	4	4
4	3	3	3
5	3	3	3
6	4	4	4
7	3	3	3
8	3	3	3
9	3	3	3
10	3	3	3
11	3	3	3
12	3	3	3
13	3	3	3
14	4	4	4
15	4	4	4
16	4	4	4
17	3	3	3
18	4	4	4
19	4	4	4
20	4	4	4
21	4	4	4
22	3	3	3
Total average score for all ChatGPT responses			3.41

**Figure 1:**
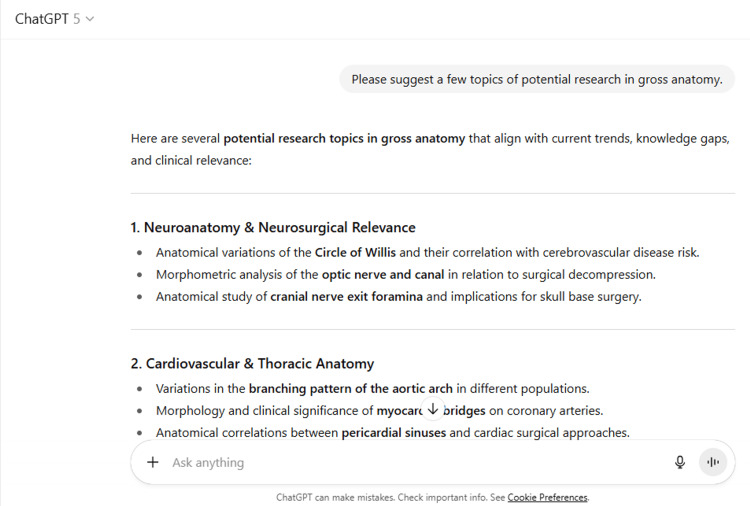
Topics of potential research in gross anatomy created by ChatGPT.

**Figure 2:**
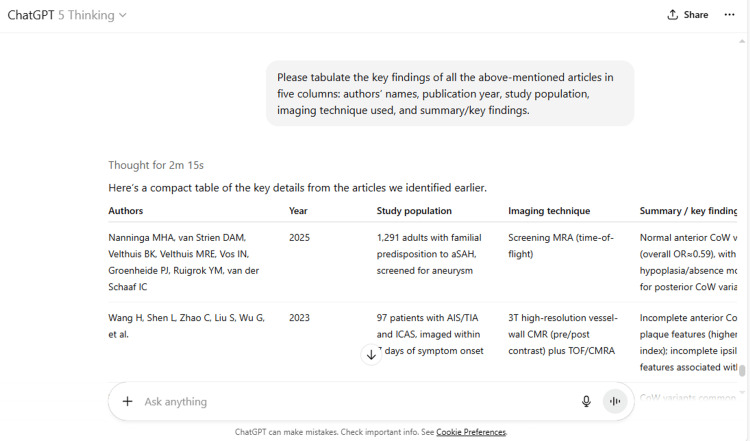
Key findings of articles tabulated by ChatGPT. aSAH: aneurysmal subarachnoid hemorrhage; AIS: acute ischemic stroke; TIA: transient ischemic attack; ICAS: intracranial atherosclerotic stenosis; MRA: magnetic resonance angiogram; CMR: cardiovascular magnetic resonance; TOF/CMRA: time-of-flight/coronary magnetic resonance angiography; CoW: circle of Willis

**Figure 3:**
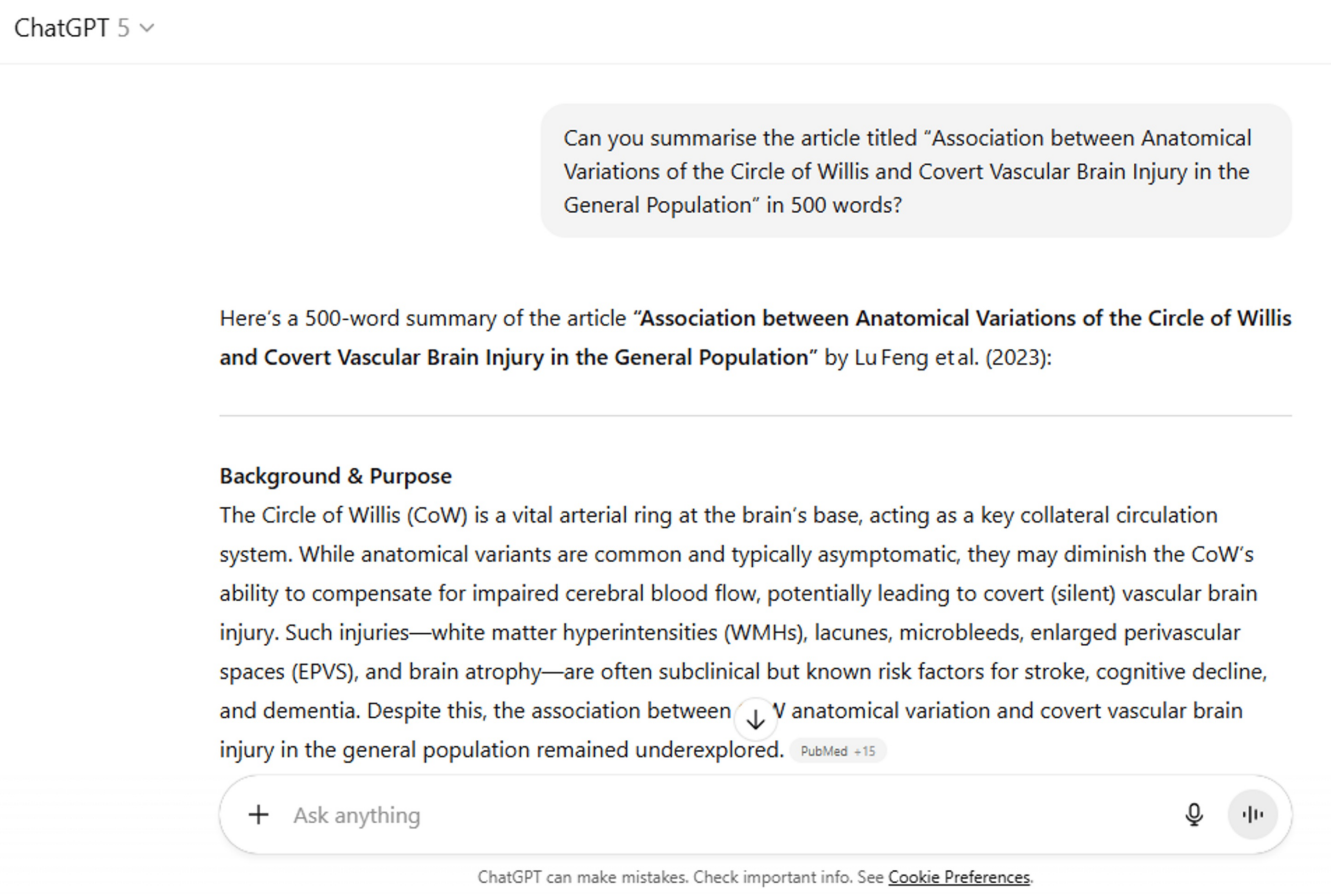
Summary of the article generated by ChatGPT.

**Figure 4:**
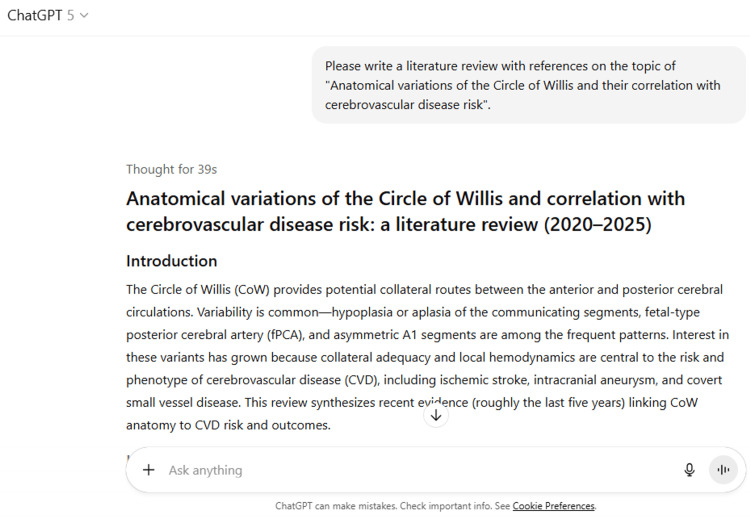
Literature review produced by ChatGPT.

**Figure 5:**
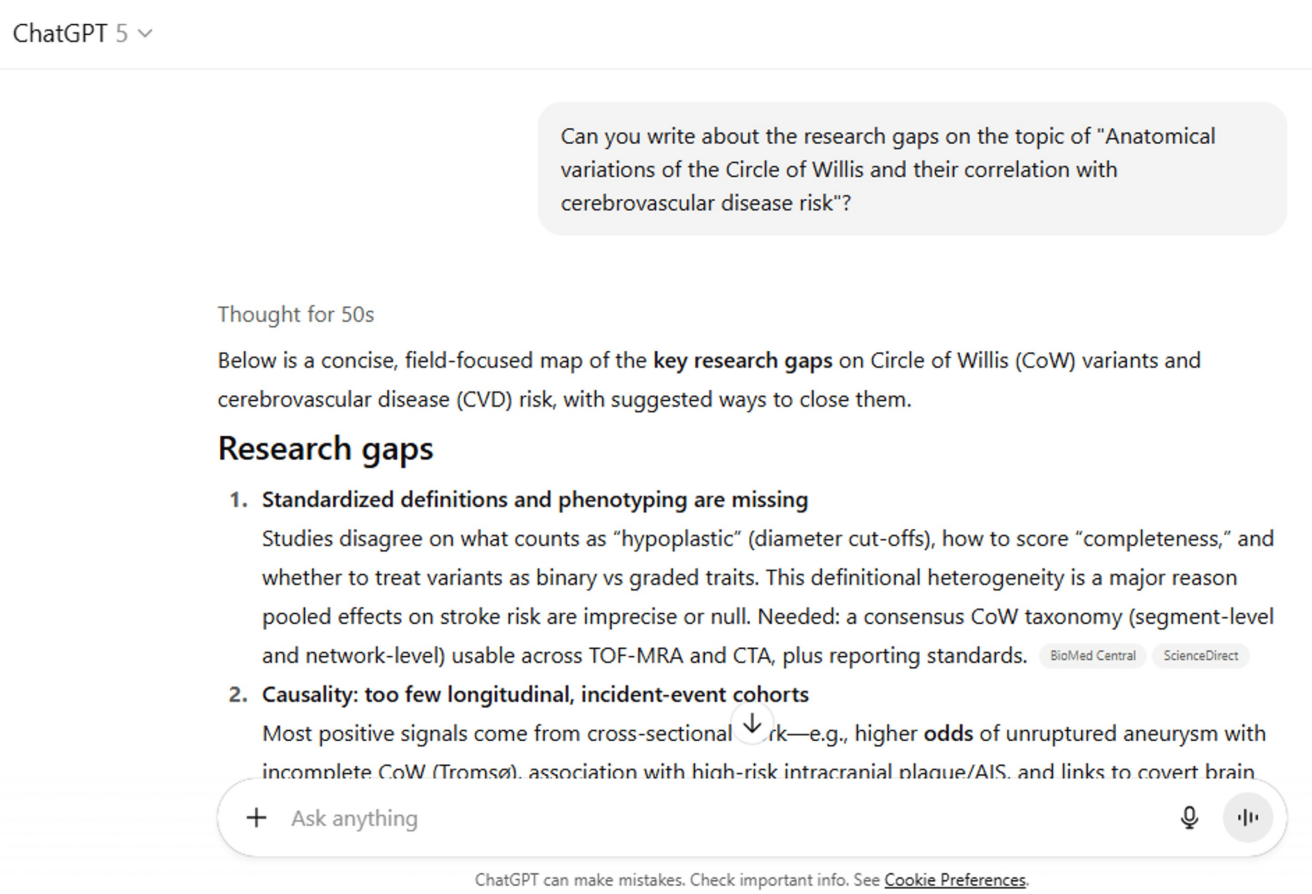
Research gaps written by ChatGPT. TOF-MRA: time-of-flight magnetic resonance angiography; CTA: computed tomography angiography

**Figure 6:**
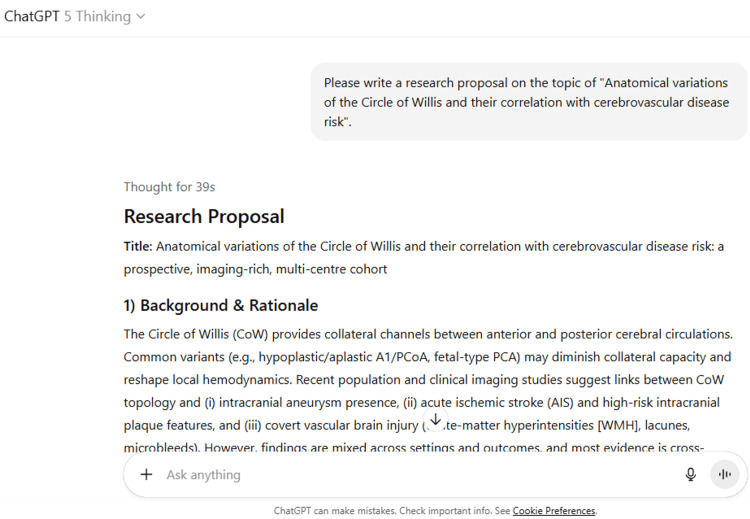
Research proposal formed by ChatGPT. PCoA: posterior communicating artery; PCA: posterior cerebral artery

**Figure 7:**
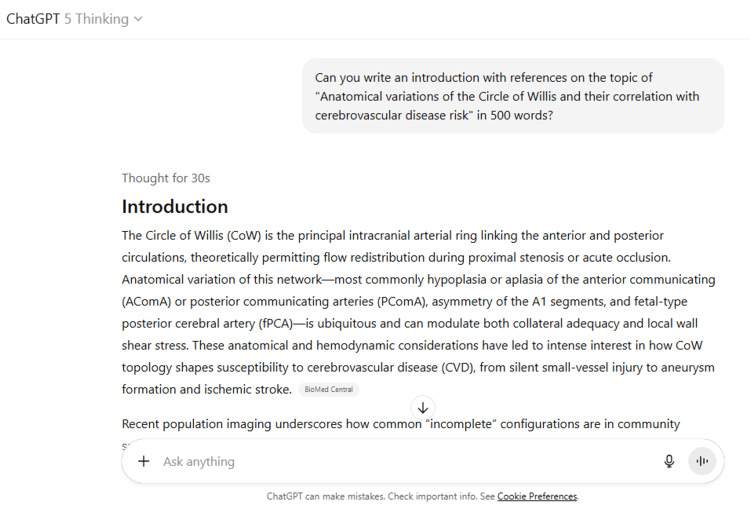
Introduction made by ChatGPT.

**Figure 8:**
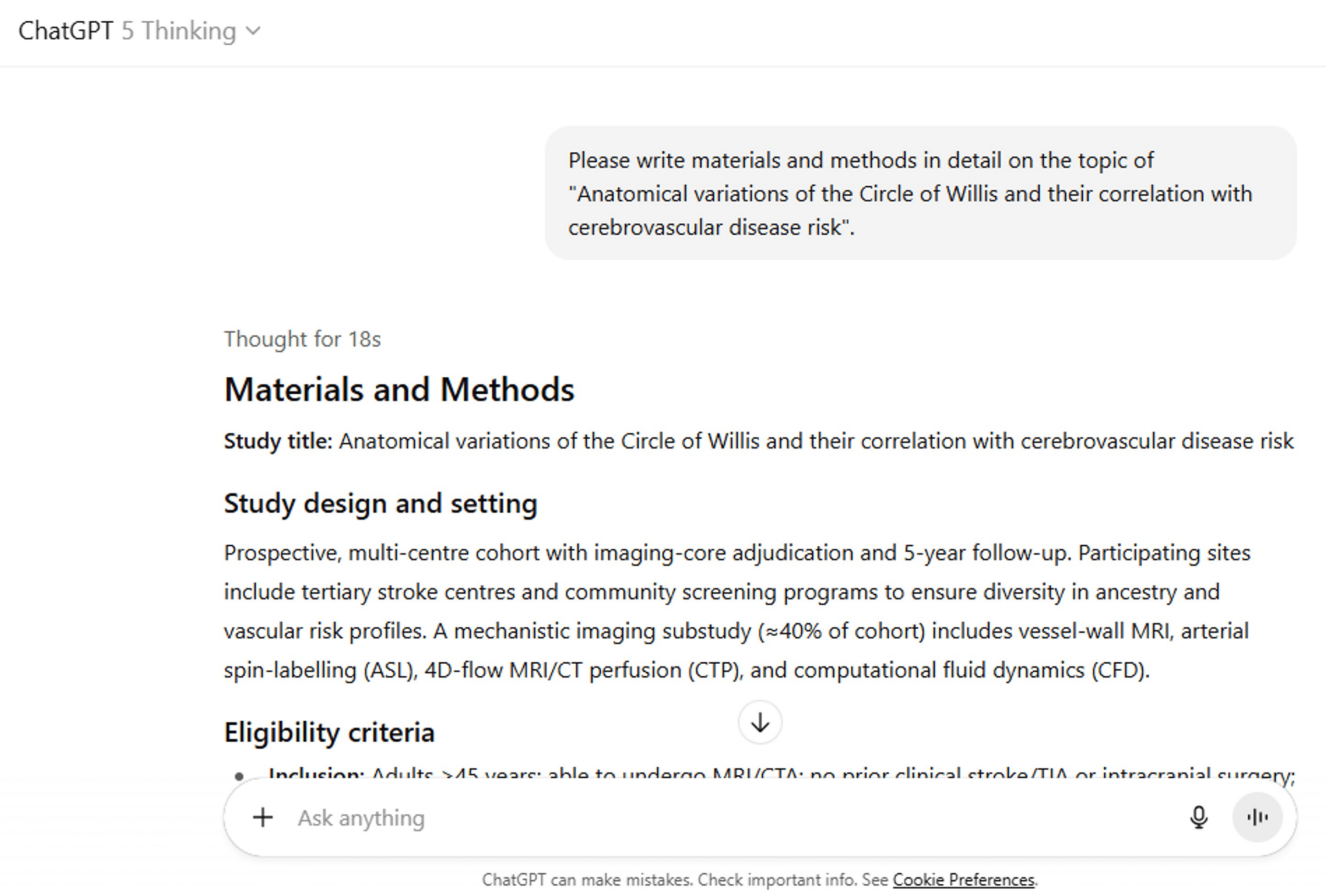
Materials and methods formulated by ChatGPT.

**Figure 9:**
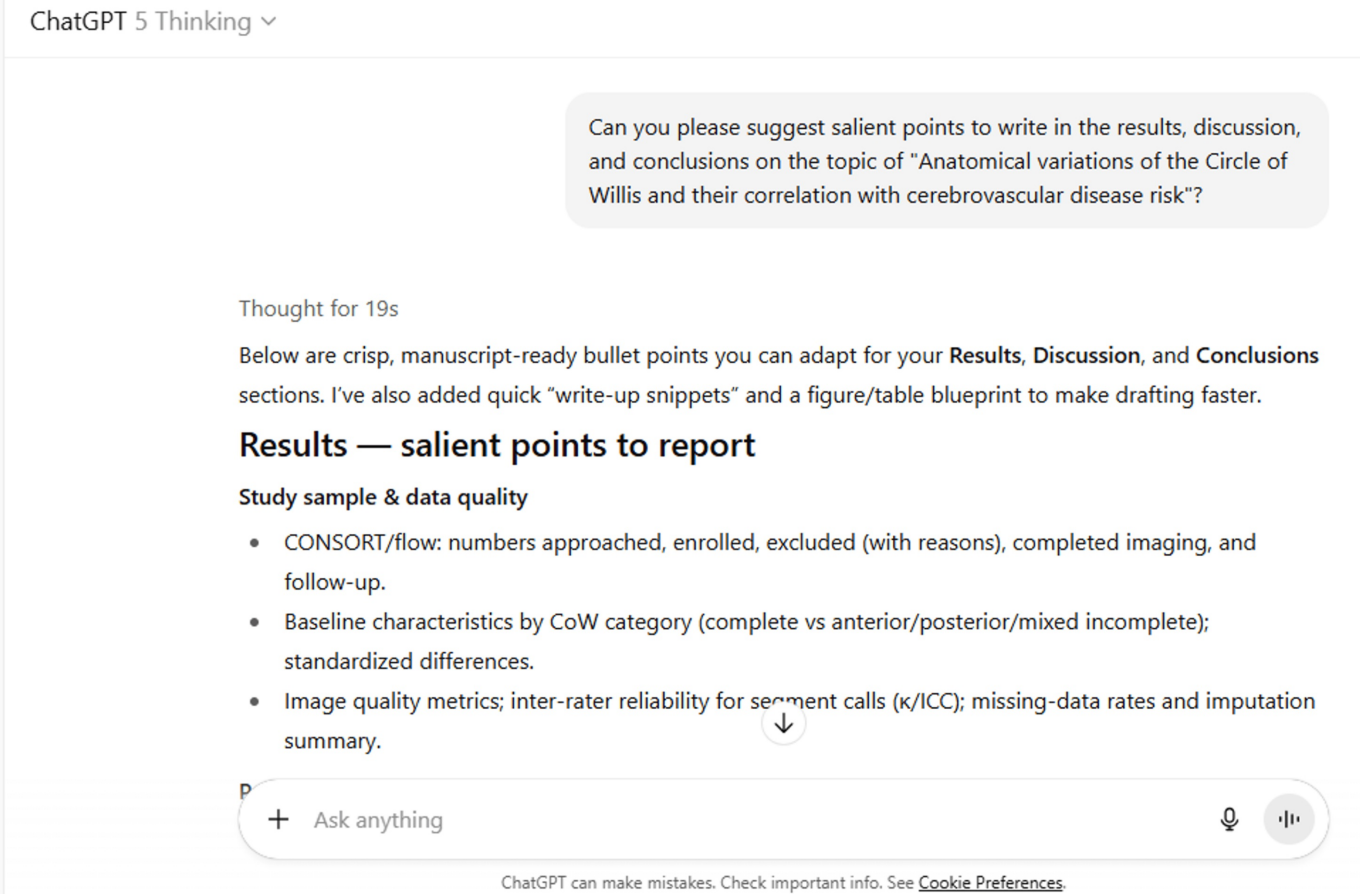
Salient points to write in results, discussion, and conclusions suggested by ChatGPT.

ChatGPT suggested several topics of potential research in anatomy based on clinical relevance, novelty, feasibility, and quantifiable outcomes (Figure 1 and Table 3). The chatbot nicely compiled a list of articles with DOIs published in high-impact journals over the last five years on the provided topic and also tabulated their key findings (Figure 2 and Table 3). It generated a fine summary of an article, informed about the research gaps, and wrote a good literature review as well as a research proposal on the given topic (Figures 3-6 and Table 3). It was noticed to provide assistance to the researcher in the preparation of the manuscript during the writing of the introduction, materials and methods, results, discussion, and conclusions (Figures 7-9 and Table 3).

**Figure 10:**
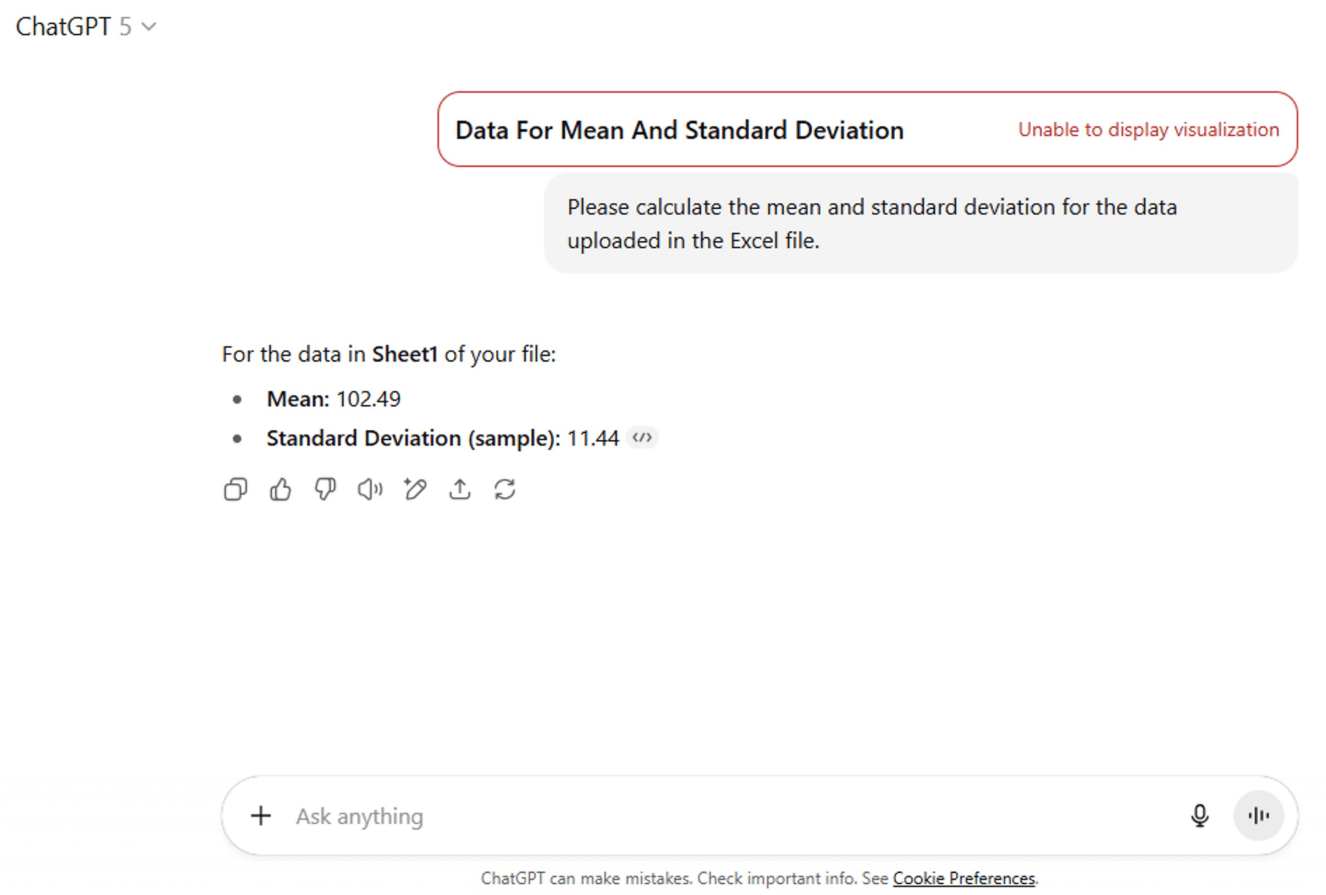
Mean and standard deviation calculated by ChatGPT.

**Figure 11:**
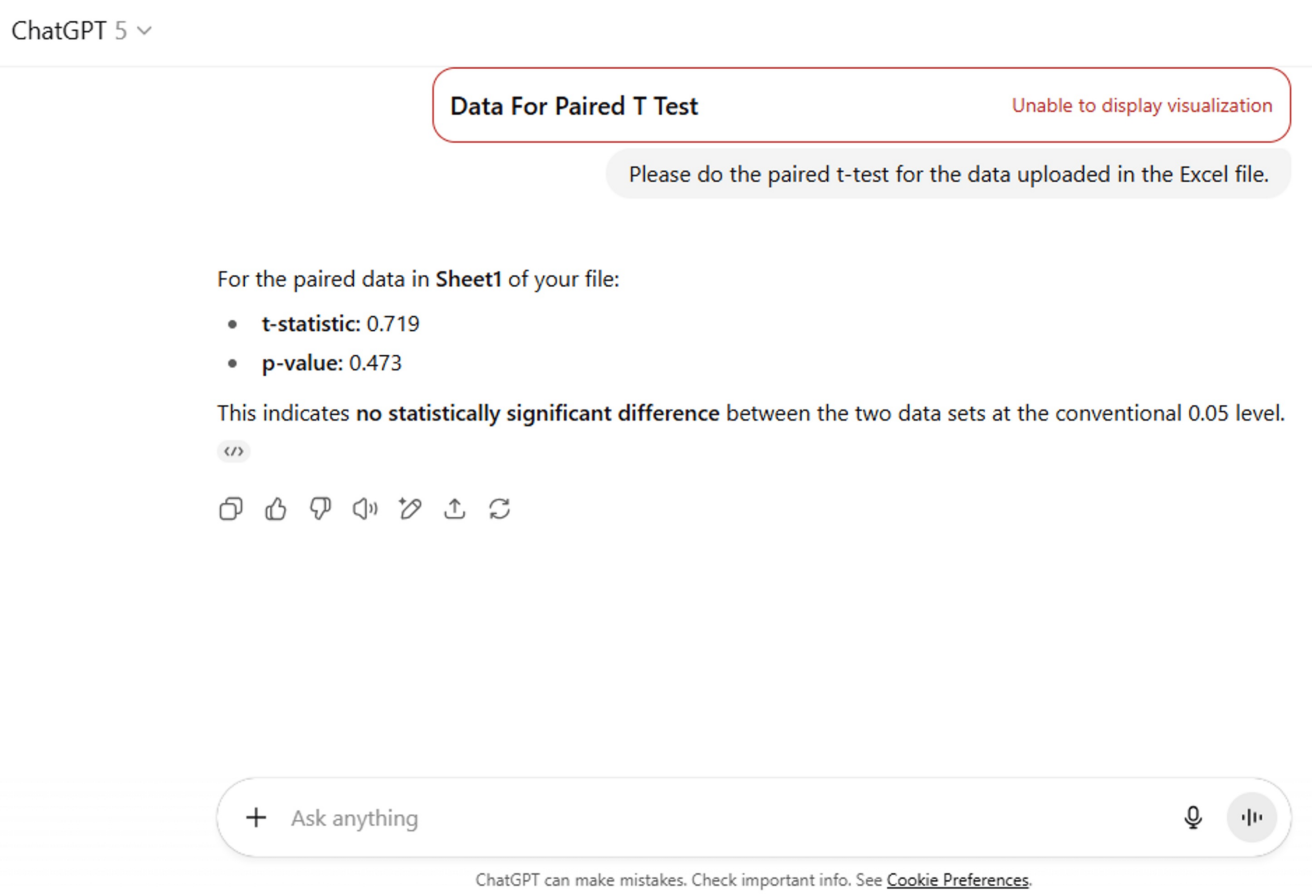
Paired t-test performed by ChatGPT.

**Figure 12:**
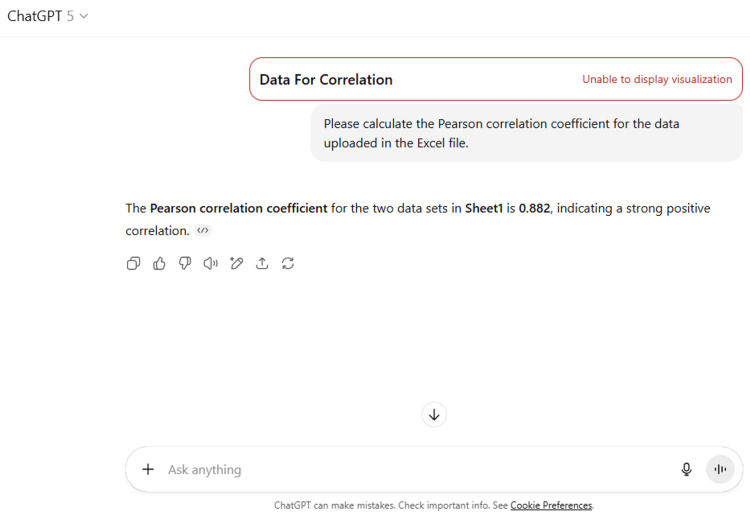
Pearson correlation coefficient calculated by ChatGPT.

**Figure 13:**
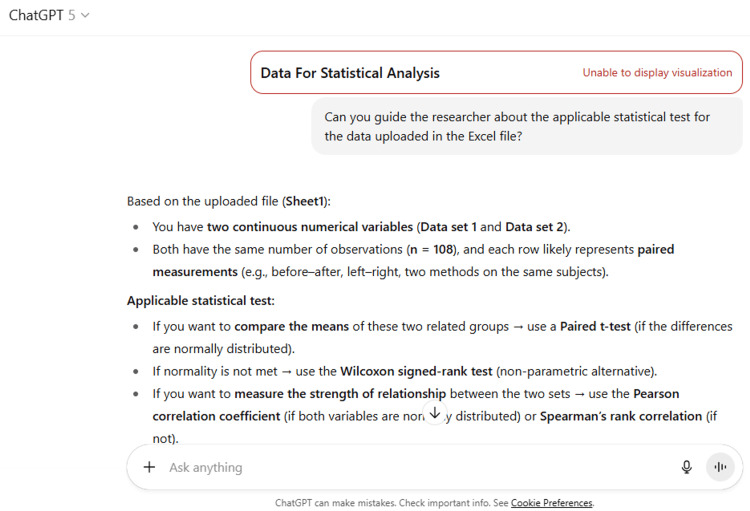
Statistical tests suggested by ChatGPT.

**Figure 14:**
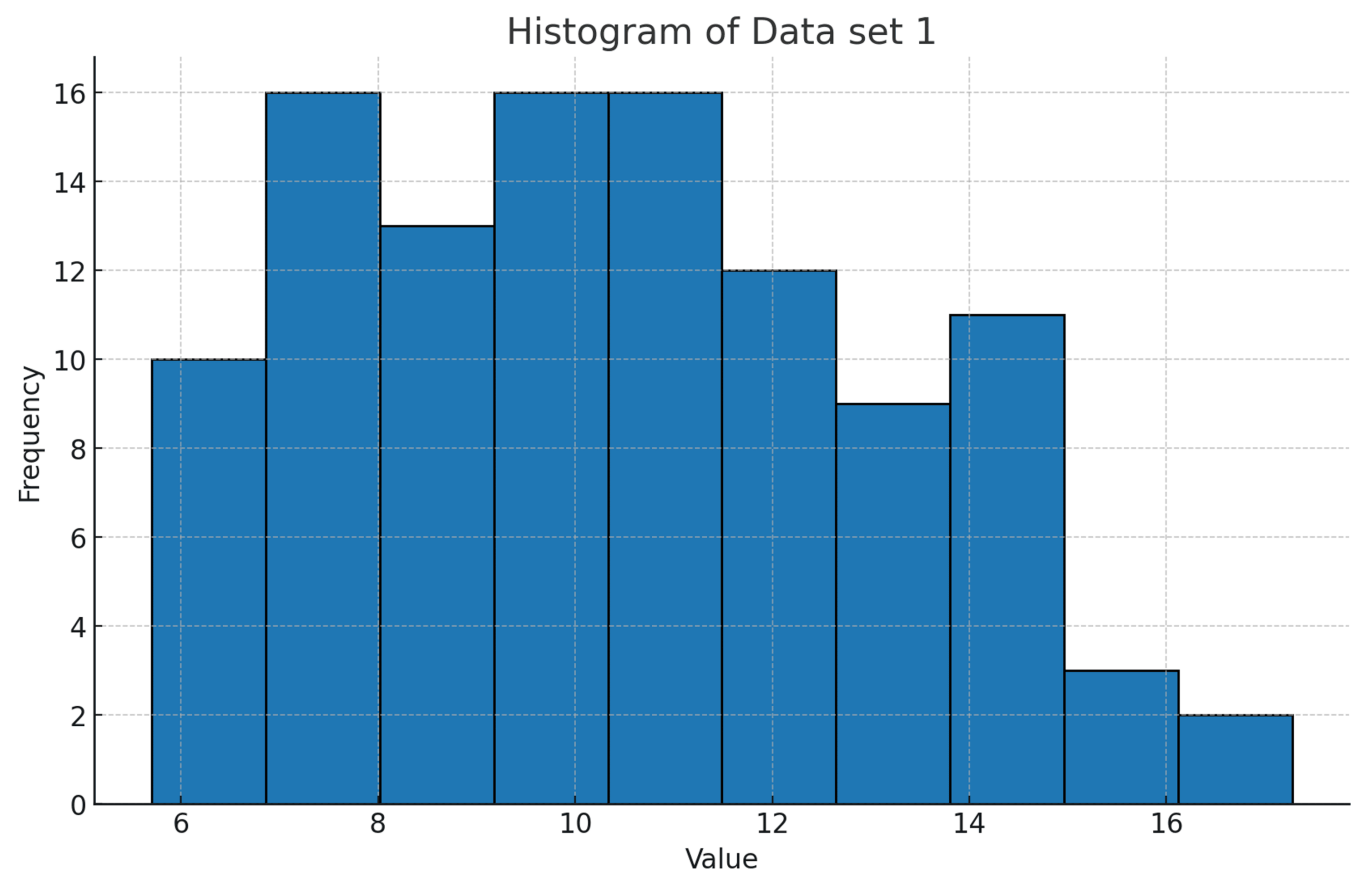
Histogram made by ChatGPT. Data set 1 represents the height of the right first posterior sacral foramina of 108 dry adult human sacra (in mm).

**Figure 15:**
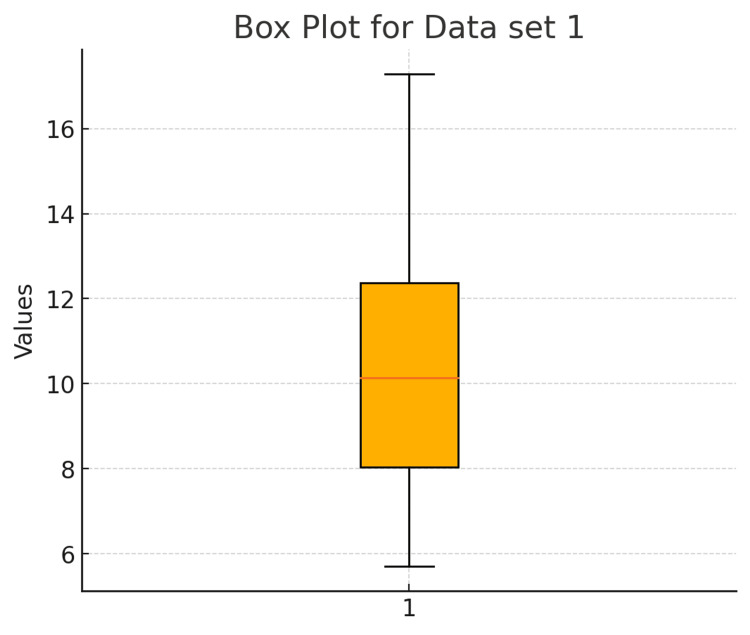
Box plot created by ChatGPT. Data set 1 represents the height of the right first posterior sacral foramina of 108 dry adult human sacra (in mm).

**Figure 16:**
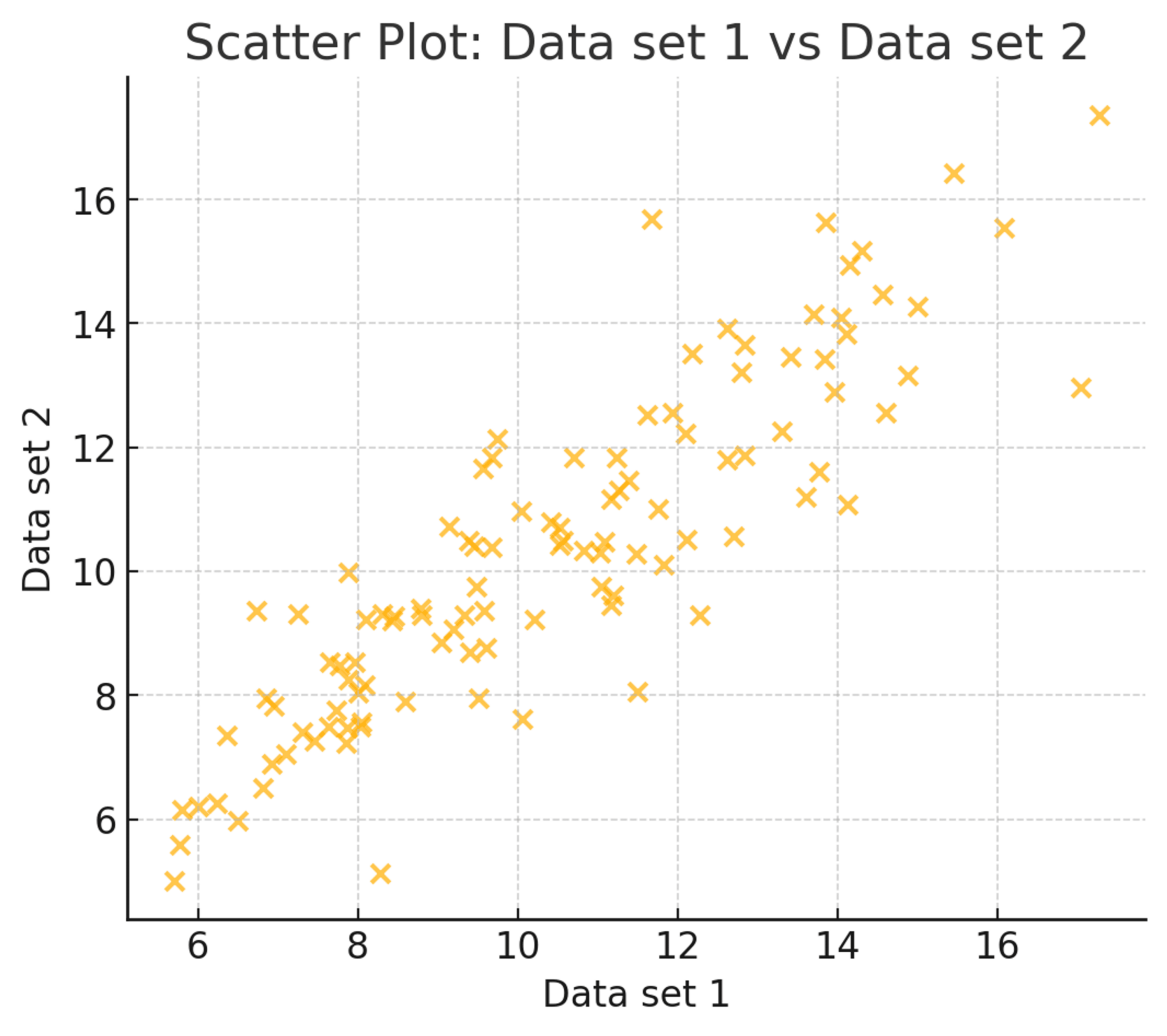
Scatter plot drawn by ChatGPT. Data set 1 represents the height of the right first posterior sacral foramina and data set 2 represents the height of the left first posterior sacral foramina of 108 dry adult human sacra (in mm).

**Figure 17:**
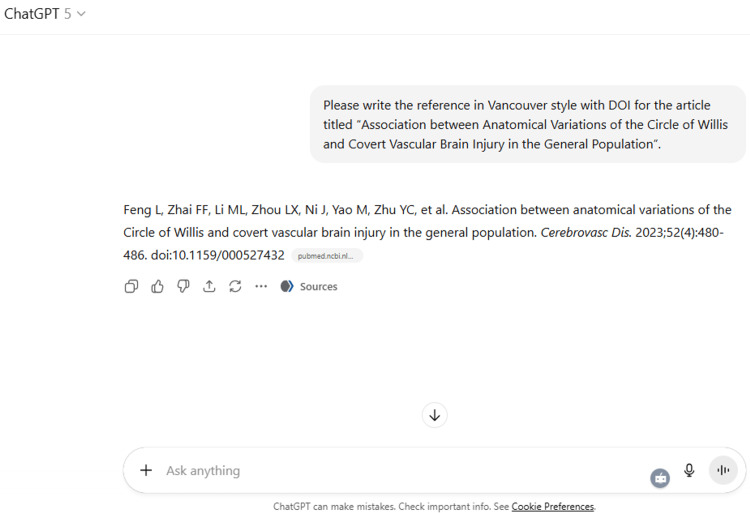
Reference of the article written in Vancouver style by ChatGPT.

**Figure 18:**
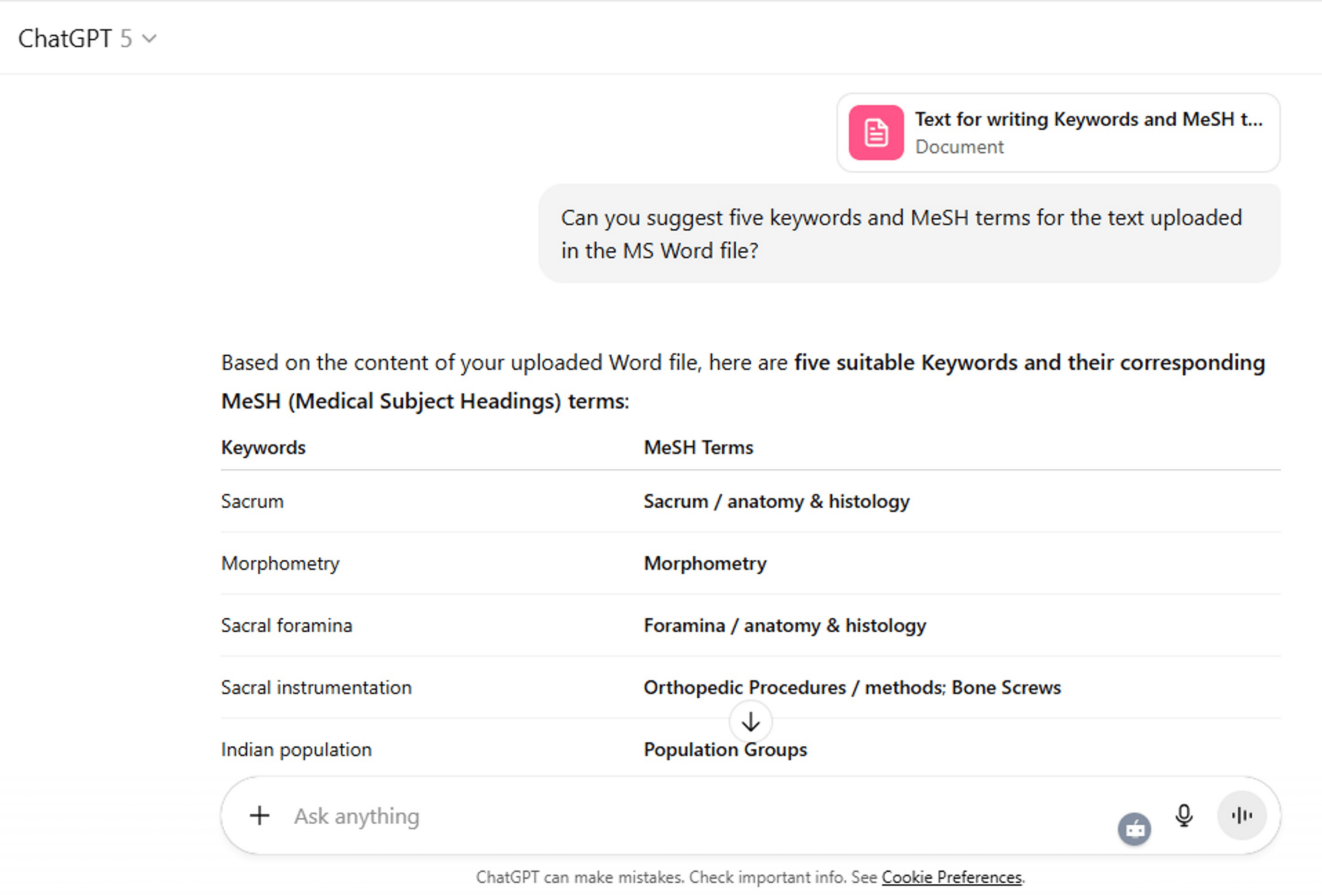
Keywords and MeSH terms suggested by ChatGPT. MeSH: Medical Subject Headings

ChatGPT was found to be quite capable of providing guidance about the applicable statistical test for any given data, and it also performed various statistical tests, including the calculation of mean, standard deviation, Pearson correlation coefficient, and Student's t-test correctly (Figures 10-13 and Table 3). Furthermore, it offered aid in the graphical visualisation of the data by drawing histograms, box plots, and scatter plots (Figures 14-16 and Table 3). Additionally, the chatbot was observed to assist in writing the references for the articles in the preferred style, specifically including the Vancouver referencing style (Figure 17 and Table 3). It also generated good keywords and their comparable Medical Subject Headings (MeSH) terms for the text uploaded in the MS Word file (Figure 18 and Table 3).

**Figure 19:**
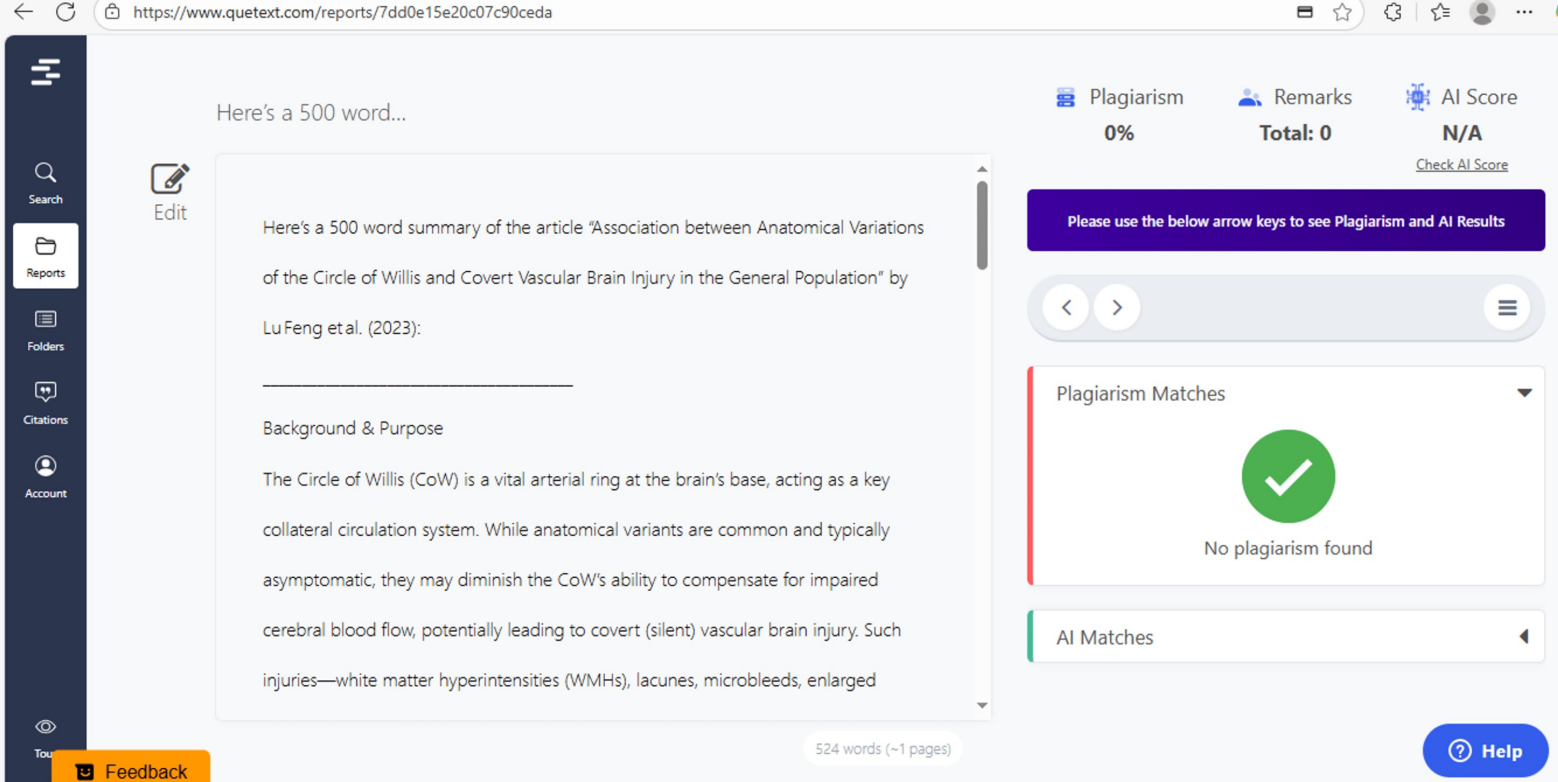
Report of Quetext to ChatGPT’s response for prompt number 6.

Ultimately, after assessment of all the responses of ChatGPT by the Quetext online plagiarism detection tool, plagiarism by the chatbot was found to be in the range of 0-96% (Figure 19 and Table 5).

**Table 5:** Report of Quetext to ChatGPT's responses.

ChatGPT's response number	Report of Quetext
1	0% overall similarity
2	3% overall similarity
3	0% overall similarity
4	44% overall similarity
5	5% overall similarity
6	0% overall similarity
7	11% overall similarity
8	0% overall similarity
9	7% overall similarity
10	0% overall similarity
11	3% overall similarity
12	0% overall similarity
13	5% overall similarity
14	Report was not generated by Quetext, as it requires a minimum 20 words to search for plagiarism
15	41% overall similarity
16	Report was not generated by Quetext, as it requires a minimum 20 words to search for plagiarism
17	8% overall similarity
18	Report was not generated by Quetext, as it requires a minimum 20 words to search for plagiarism
19	0% overall similarity
20	Report was not generated by Quetext, as it requires a minimum 20 words to search for plagiarism
21	96% overall similarity
22	0% overall similarity

## Discussion

ChatGPT is a valuable tool which can provide aid to investigators in multiple facets of the research endeavour, encompassing literature review, data analysis, and step-by-step drafting of a scientific paper [11]. The present study submitted a few prompts to ChatGPT to evaluate its current potential in AR and writing a scientific paper.

We started our interaction with ChatGPT by enquiring about its role in AR and publication. The chatbot described its capability in AR spanning multiple stages of the scientific process, encompassing topic selection, literature review, data analysis, manuscript preparation, and reference management. Further, we asked ChatGPT to suggest some topics of potential research in gross anatomy, and it responded by presenting fine topics aligning with current trends involving neuroanatomy, cardiovascular, musculoskeletal, abdominal and pelvic, head and neck, and forensic and anthropological anatomy (Figure 1). The chatbot selected the research topics using criteria that balance academic novelty, clinical relevance, feasibility, publication potential, interdisciplinary appeal, and quantifiable outcome. Similar to our findings, Chukwuere observed that ChatGPT was an important tool for quick research topic generation [11]. Thereafter, we selected a topic from the list generated by ChatGPT and instructed it to compile a list of articles with DOIs. The chatbot accurately produced a list of valid, latest, clinically relevant articles with their exact DOI published in high-impact journals over the last five years. Additionally, it also created a good table from all these articles showing the key findings, including authors' names, publication year, study population, imaging technique used, and summary (Figure 2 and Table 3). 

When asked to summarise an article in 500 words, ChatGPT created an up-to-the-mark summary incorporating background, purpose, study population, methodology, key findings, discussion, limitations, and conclusions (Figure 3). Further, we instructed ChatGPT to write a literature review on the topic of "Anatomical variations of the Circle of Willis and their correlation with cerebrovascular disease risk". It created a fair literature review with references involving the circle of Willis variations in covert vascular brain injury, intracranial aneurysm risk, large artery atherosclerosis, and acute ischemic stroke (Figure 4). In the same way, Biswas, in his study on ChatGPT, observed that ChatGPT can generate a basic literature review on a given topic which can be modified by the authors [7]. Further, we requested ChatGPT to write research gaps on the same topic. The chatbot meticulously delineated the research gaps on the circle of Willis variants and cerebrovascular disease risk, and it also offered suggestions to close them (Figure 5 and Table 3). Comparable to our findings, Chukwuere noticed that ChatGPT can help researchers identify knowledge gaps and suggest topics for further research by thoroughly exploring relevant scholarly literature [11].

Considering the importance of submission of a plan prior to conducting the research, we instructed ChatGPT to write a research proposal on the same topic, and it generated a well-structured prospective cohort research proposal with all necessary components, including study objectives, design, statistical analysis plan, data quality, ethical considerations, timeline, feasibility, risks, and expected impact (Figure 6 and Table 3).

Thereafter, we requested ChatGPT to write an introduction and methodology on the topic of "Anatomical variations of the Circle of Willis and their correlation with cerebrovascular disease risk". It formulated a decent introduction highlighting the background, current knowledge gaps, and rationale of the study (Figure 7). Likewise, Biswas also found that ChatGPT generated a fair introduction, including the objectives of the research, which can then be revised by the investigator [7]. Furthermore, the chatbot also generated a good methodology encompassing detailed study design, eligibility criteria, consent, baseline assessment, imaging protocols, follow-up schedule, data management, sample size consideration, statistical analysis, and ethical consideration (Figure 8). In the same way, Biswas noticed that ChatGPT can help in writing methodology by providing recommendations and guidance based on the input of the title by the investigator [7]. Subsequently, ChatGPT was directed to provide salient points to write the results, discussion, and conclusions on the same topic. It provided adequate key points to draft the results, including the prevalence of circle of Willis variants, MRI markers of covert cerebrovascular injury, clinical endpoints, laterality, territory, mediation, and model performance. The chatbot also suggested main points to prepare in writing the discussion and conclusions incorporating principal findings, biological plausibility, context, clinical implications, strengths, limitations, and future directions (Figure 9 and Table 3). Likewise, Biswas noted that ChatGPT can serve as a useful aid in generating a thorough and well-formed discussion and conclusion for research papers [7]. Al-Sofi also observed that ChatGPT was helpful for creating appropriate texts for research work, summarising literature, improving research papers, and assisting with proofreading [12].

Furthermore, to assess the capability of ChatGPT to perform statistical analysis, we uploaded the Excel file containing arbitrary data and instructed it to determine the mean, standard deviation, Pearson correlation coefficient, and paired t-test. The chatbot analysed the data accurately and provided the exact value of these statistical tests (Figures 10-12). Comparable to our observations, Chukwuere and AlZaabi et al. noticed that ChatGPT holds the potential to offer support to investigators for the purpose of statistical analysis and interpretation of research data [11,13]. Subsequently, to evaluate the potential of ChatGPT in the graphical visualisation of a data set, we instructed it to draw the histogram, box plot, and scatter plot after uploading the data in the Excel file, and the chatbot was observed to illustrate the data precisely in various modes of representation (Figures 14-16).

Thereafter, we requested the chatbot to write the reference with the DOI of an article in Vancouver style, and it generated an accurate citation in the preferred format (Figure 17 and Table 3). Moreover, we observed that ChatGPT produced precise article references when requested to mention their DOI also. In the same way, Huang and Tan noticed that ChatGPT can generate the appropriate citations in addition to its role in topic selection and literature review [14]. On the one hand, Biswas observed that most of the references created by ChatGPT were erroneous, but it was able to modify the references into any desired manner [7]. On the other hand, Mondal and Mondal noticed that ChatGPT could be utilised for the preparation of references, but it should be manually confirmed and validated [15].

Ultimately, we instructed ChatGPT to suggest keywords and MeSH terms for the uploaded text in the MS Word file, and it created excellent keywords and their corresponding MeSH terms (Figure 18 and Table 3). Similar findings were observed by Biswas in his study on ChatGPT for research and publication [7].

Various researchers have reported that ChatGPT might unintentionally produce text that resembles or reproduces existing content without appropriate citation or acknowledgement [3,7,8,11]. Therefore, to evaluate the plagiarism by ChatGPT, we submitted all the responses of the chatbot to the Quetext online plagiarism detection tool and found the plagiarism in the range of 0-96%, depending upon the type of content scrutinised. The responses produced by the chatbot displayed overall plagiarism of up to 11% for tabulating the key findings of the various articles, generating the summary of an article, writing a literature review, and writing a research proposal. However, it exhibited 44% plagiarism for compiling a list of articles with a DOI and 96% plagiarism for writing an article in Vancouver referencing style, which can be understood and justified (Table 5).

Hence, it is proposed that ChatGPT can be utilised as a good assistant in AR with capabilities of suggesting research topics, writing literature reviews and research proposals, analysing the data, and preparing the manuscript with minimal risk of plagiarism if utilised appropriately.

The current study submitted only a few prompts to assess ChatGPT's potential role in AR. Therefore, the responses of the chatbot may vary if researchers explore various other prompts for a wide range of anatomical structures. We advocate additional investigations to offer recommendations for the best possible employment of ChatGPT in AR.

## Conclusions

ChatGPT can be a valuable tool for AR and has the capability to reshape academic research and writing by enhancing interaction, constructiveness, information access, and creativity. It can appropriately suggest the topics of potential AR, compile and tabulate the key findings of the preferred articles, write the literature review and research proposal, help in manuscript preparation, and do the statistical analysis and graphical visualisation of the data. 
